# Effects of simulated drought on biological soil quality, microbial diversity and yields under long-term conventional and organic agriculture

**DOI:** 10.1093/femsec/fiaa205

**Published:** 2020-10-05

**Authors:** Dominika Kundel, Natacha Bodenhausen, Helene Bracht Jørgensen, Jaak Truu, Klaus Birkhofer, Katarina Hedlund, Paul Mäder, Andreas Fliessbach

**Affiliations:** Department of Soil Sciences, Research Institute of Organic Agriculture (FiBL), 5070 Frick, Switzerland; Ecology, Department of Biology, University of Konstanz, 78464 Konstanz, Germany; Department of Soil Sciences, Research Institute of Organic Agriculture (FiBL), 5070 Frick, Switzerland; Department of Biology, Lund University, 221 00 Lund, Sweden; Institute of Molecular and Cell Biology, University of Tartu, 51010 Tartu, Estonia; Department of Ecology, Brandenburg University of Technology, 03046 Cottbus, Germany; Department of Biology, Lund University, 221 00 Lund, Sweden; Department of Soil Sciences, Research Institute of Organic Agriculture (FiBL), 5070 Frick, Switzerland; Department of Soil Sciences, Research Institute of Organic Agriculture (FiBL), 5070 Frick, Switzerland

**Keywords:** microbial diversity, rainout shelter, PLFA, amplicon-based sequencing, DOK trial, soil organic carbon

## Abstract

Drought and agricultural management influence soil microorganisms with unknown consequences for the functioning of agroecosystems. We simulated drought periods in organic (biodynamic) and conventional wheat fields and monitored effects on soil water content, microorganisms and crops. Above the wilting point, water content and microbial respiration were higher under biodynamic than conventional farming. Highest bacterial and fungal abundances were found in biodynamically managed soils, and distinct microbial communities characterised the farming systems. Most biological soil quality parameters and crop yields were only marginally affected by the experimental drought, except for arbuscular mycorrhizal fungi (AMF), which increased in abundance under the experimental drought in both farming systems. AMF were further strongly promoted by biodynamic farming resulting in almost three times higher AMF abundance under experimental drought in the biodynamic compared with the conventional farming system. Our data suggest an improved water storage capacity under biodynamic farming and confirms positive effects of biodynamic farming on biological soil quality. The interactive effects of the farming system and drought may further be investigated under more substantial droughts. Given the importance of AMF for the plant's water supply, more in-depth studies on AMF may help to clarify their role for yields under conditions predicted by future climate scenarios.

## Teaser

The impact of simulated summer drought on biological soil properties, microbial diversity and winter wheat yields was compared with rainout shelters under long-term conventional and organic agriculture.

## INTRODUCTION

Soil microorganisms are of crucial importance for soil functions and the provisioning of ecosystem services (Bardgett and Van Der Putten 2014; Nielsen, Wall and Six 2015). Abiotic stressors, such as severe droughts and intensive agricultural management, may negatively influence the abundance, diversity and functioning of microbial communities (Cavicchioli *et al*. 2019). Climate models foresee increasingly frequent and severe droughts in southern and most of central Europe along with reduced amounts of summer precipitation (Pachauri *et al*. 2014). Given these projected climatic conditions, it is a research priority to understand how soil microorganisms react to drought.

The effect of drought on microbial communities is complex since it is modified by numerous factors, including the frequency, intensity and duration of the drought, the impact of drought on higher-trophic-level soil organisms, resource preferences of microorganisms and their specific adaptive potential (Naylor and Coleman-Derr 2018; Schimel 2018). Furthermore, in the agricultural context, soil and crop management may influence the ultimate effects of droughts. More specifically, the effects of drought on soil microorganisms may differ between organic and conventional farming systems. These systems follow profoundly different concepts of fertilisation and crop protection, which in turn influence physical, chemical and biological soil properties (Mäder *et al*. 2002; Birkhofer *et al*. 2008; Lori *et al*. 2017). Soils in organically managed fields are, for example, characterised by higher levels of soil organic carbon (SOC) compared with soils under conventional farming with mineral fertilisers (Gattinger *et al*. 2012). High levels of SOC can improve the soil structure, thereby enhancing water infiltration and soil water retention (Rawls *et al*. 2003; Huntington 2006) and may ultimately buffer soil organisms from being exposed to drought.

Organically managed soils with high SOC levels are further characterised by higher microbial abundance, activity (Lori *et al*. 2017) and increased microbial diversity (Hartmann *et al*. 2015; Harkes *et al*. 2019). The resistance of microbial communities to disturbances is suggested to be linked to diversity (Bardgett and Caruso 2020) because diversity can increase the variability of species’ responses to environmental stress and may thus buffer essential ecosystem functions against environmental fluctuations (Naeem and Li 1997; Yachi and Loreau 1999). Under controlled conditions, Lori *et al*. (2018) found evidence for a more stable nitrogen provisioning under drought, which correlated closely to the higher functional gene diversity of the underlying proteolytic community in soils under organic compared to conventional farming. Moreover, plant biomass production under drought was less impaired under organic compared to conventional management (Lori *et al*. 2018). While this study underpins the role of local soil properties and agricultural management in the context of a controlled drought, this needs to be proven under field conditions. To date, most field experiments have explored how agricultural management can modulate the effects of experimental drought on microbial communities in grasslands (De Vries *et al*. 2012; Karlowsky *et al*. 2018; Fuchslueger *et al*. 2019; Siebert *et al*. 2019) but not in arable production systems with annual crops (cereals).

Here, we report on the results of a field experiment in which we simultaneously studied the effects of experimental drought and agricultural management on soil water content, properties of microbial communities, crop growth and yields. We conducted the study in the DOK experiment, one of the oldest farming system comparison trials worldwide (Mäder *et al*. 2002; Krause *et al*. 2020), using replicated field plots of the biodynamic and conventional farming systems under winter wheat production. The soils in the biodynamic and conventional farming systems are known to differ in physical, chemical and biological soil parameters (Mäder *et al*. 2002; Fliessbach *et al*. 2007; Birkhofer *et al*. 2008) and therefore allowed to study the effects of drought under contrasting agricultural management and soil properties, respectively. In both farming systems, we established rainout shelters along with controls (Kundel *et al*. 2018) and analysed microbial properties together with basic soil characteristics and plant traits, including above- and belowground biomass production. To understand the dynamics of responses to summer drought, we took the majority of measurements at three sampling dates across the main growing season.

Based on the above-mentioned contrasting physical and biological soil characteristics resulting from different soil management practices, we expected to find a higher soil water content in the biodynamic compared with the conventional farming system. Furthermore, we assumed that the experimental drought changes microbial respiration, diversity and community composition, especially under conventional management. Given the complex interactions between microbes and drought-induced effects on the plant and other members of the soil food web, we did not have an *a priori* hypothesis regarding the effect of drought on bacterial and fungal abundance. Finally, we expected that the experimental drought would have adverse effects on the plant (root biomass, cereal and straw yields), especially under conventional management.

## MATERIALS AND METHODS

### Study site

The study was performed in 2017 using the ‘DOK’ trial, a long-term farming system comparison trial (Therwil, Switzerland, 47°30′09.3″N, 7°32′21.5″E) as a platform for the current study. The DOK trial, established in 1978, has compared agricultural production in organic and conventional farming systems following the same 7-year crop rotation (Mäder *et al*. 2002; Krause *et al*. 2020). The site is situated at 300 m above sea level and has a slope of 3–5% in South–North direction. The soil is a Haplic Luvisol on deep sediments of loess (Fliessbach *et al*. 2007). Averaged over the last 5 years, the mean annual temperature at the site was 10.5°C, and the mean annual precipitation was 890 mm (https://www.bodenmessnetz.ch/messwerte/datenabfrage; data retrieved on 1 August 2019). The study was performed in plots of winter wheat (*Triticum aestivum* L. cv. ‘Wiwa’) of the biodynamic and conventional farming systems (hereafter referred to as the factor system) with soybeans as preceding crop. In the last 40 years, the biodynamic farming system (hereafter called BioDyn) has been managed according to the guidelines for ‘Demeter’ food production (https://demeter.ch/). The BioDyn system shares many characteristics with the organic farming system, e.g. it relies exclusively on organic fertilisation (slurry, composted animal manure) and biological pest and mechanical weed control but on top applies biodynamic preparations to soils, plant and compost (Mäder *et al*. 2002). The conventional farming system (hereafter called ConMin), apart from senescent crop residues, has been receiving only (synthetic) mineral fertiliser according to Swiss guidelines (Richner & Sinaj 2017) next to insecticides, herbicides and fungicides following principles of integrated production systems (IP-SUISSE). An overview of our management operations is provided (Table S1, Supporting Information).

### Experimental design

In each of the four replicate plots per farming system (5 m × 20 m), natural precipitation levels were manipulated (hereafter called the factor drought) by setting up fixed-location, partial rainout shelters (2.5 m × 2.5 m) along with two control treatments, resulting in 24 experimental subplots (four replicate plots in two farming systems with three drought treatment subplots each). The three subplots with the drought treatments were (i) a partial rainout shelter, in which precipitation was reduced by 65% (roof), (ii) a rainout shelter control (roof control), which allowed the rain to pass, but took account of rainout shelter artefacts and (iii) an open control subplot without a shelter (control). A schematic drawing of the shelters is provided in Figure S1 (Supporting Information) and a detailed description of the rainout shelters, including their side effects on soil and air temperature, can be found in Kundel *et al*. (2018). The shelters were set up in mid-March at tillering and the experiment ended in June 2017, shortly before winter wheat harvest. Daily precipitation data were obtained from the on-field meteorological station (Campbell-CR1000) or a nearby backup station in Therwil, Switzerland (http://www.bodenmessnetz.ch/messwerte/datenabfrage).

### Sampling procedure and measurements

Before starting the experiment, undisturbed soil samples were taken from depths of 0–10 and 10–20 cm to assess bulk density and water holding capacity. In addition, disturbed soil samples were taken with a soil auger (3 cm Ø) from the top 20 cm of soil to obtain ∼1 kg of soil in which levels of soil pH, total soil carbon and nitrogen, phosphorous and phosphates, total sand, silt and clay content were assessed. Because most of these soil characteristics were assumed to remain constant throughout the season, they were determined only once.

Three other sampling campaigns were conducted in adjacent sampling areas of 0.1 m² size inside the 24 subplots. These samplings took place in April (T1), May (T2) and June (T3) corresponding to 4, 8 and 13 weeks (hereafter called the factor time) after the establishment of the drought treatments. At these sampling dates, disturbed soil samples were taken with a soil auger (3 cm Ø) between wheat rows, from the top 20 cm of soil to obtain ∼1 kg of soil. Wheat biomass was harvested (20 cm × 50 cm, two wheat rows), and four in-row root samples (5 cm Ø) were taken from the top 20 cm of soil and combined into a composite sample per subplot. On all sampling dates and in all sampling areas, plant height was measured (*n* = 4 plant per drought subplot), and the proportion of ground covered by arable weeds was visually estimated. All samples were transferred to the laboratory for further processing. Details on the conducted analyses are given in the subsequent sections.

### Soil- and plant-related analyses

Maximum water holding capacity and soil bulk density were taken from the undisturbed volumetric ring samples. For calculations of volumetric soil moisture, 100 g of fresh soil was dried at 105°C to constant weight, and soil moisture calculated on a dry weight basis considering bulk density. The field capacity and the wilting point were calculated, taking into account soil texture, bulk density and SOC content (Eckelmann *et al*. 2006).

All chemical analyses were conducted on air-dried and sieved (2 mm) soil. Soil pH was measured in water at a soil-to-water ratio of 1:2.5 (w/v), total soil carbon and nitrogen content (C_tot_, N_tot_) by an Elementar Vario Max Cube (Elementar Analysensysteme GmbH, Langenselbold, Germany). As the soil at the site is practically free of carbonates, C_tot_ is all organic carbon. Soil phosphates were determined colourimetrically (using the ascorbic acid method); phosphorus after acid digestion. Granulometric analysis (soil texture) was carried out by discontinuous sedimentation (Robinson pipette method).

Microbial abundance was assessed by phospholipid fatty acid (PLFA) analysis. Lipid extractions for PLFA analysis were made using 3 g of soil, according to Frostegård, Bååth and Tunlio (1993). The fatty acid methyl esters were separated using a Hewlett Packard 6890 gas chromatograph (Hewlett Packard, Palo Alto, USA). The sum of phospholipid fatty acids i15:0, a15:0, 15:0, i16:0, 16:1ω9, i17:0, a17:0, cy17:0, 18:1ω7, 20:0 and cy19:0 was used as an index of bacterial biomass (Frostegård and Bååth 1996), the amount of PLFA 18:2ω6 as an index of non-mycorrhizal fungal biomass and the neutral lipid fatty acid (NLFA) 16:1ω5 as a marker for arbuscular mycorrhizal fungi (AMF; Olsson *et al*. 1995). Soil basal respiration was measured according to Jäggi (1976) in field-moist soils after a 7-day pre-incubation at 22°C.

Fresh root samples were obtained by wet sieving over a 1-mm sieve until roots were free of any adherent soil. Grains were separated from straw by hand, and all plant material was dried at 60°C before weighting.

## MOLECULAR ANALYSIS

### DNA extraction

DNA was extracted from 0.25 g of homogenised and frozen soil using the DNeasy PowerSoil Kit (Qiagen, Hilden, Germany) following the manufacturer's protocols. Negative controls (DNA extraction blanks) were included. The quantity and quality of DNA extracts were determined by spectrophotometry (Infinite M200, Tecan Group Ltd, Männedorf, Switzerland); extracts were stored at −20°C until further analyses.

### Library preparation for bacterial and fungal communities

The soil prokaryotic community composition was assessed by Illumina^®^ MiSeq sequencing of combinatorial sequence-tagged polymerase chain reaction (PCR) products using the universal barcoded (Parameswaran *et al*. 2007) primers 515F and 926R to target the 16S rRNA gene region (Parada, Needham and Fuhrman 2016). Sample amplification was performed in triplicate in an Eppendorf Mastercycler PCR machine (Eppendorf, Hamburg, Germany). We included a negative control (DNA was replaced by water) in all PCR runs. Details on the PCR reaction and run condition are provided in Table S2 (Supporting Information). After the replicate PCR products were pooled, their concentration was measured with the TapeStation 2200 using D1000 ScreenTapes^®^ (Agilent Technologies, Santa Clara, CA, USA), and samples were combined in equal proportions. The final library was then purified and concentrated using the NucleoSpin^®^ Extract II kit (Macherey-Nagel GmbH & Co. KG, Düren, Germany). The paired-end DNA library was prepared by adaptor ligation and PCR using the TruSeq Nano DNA Library Prep Kit, excluding the fragmentation step (Herbold *et al*. 2015) and sequenced on an Illumina^®^ MiSeq system (2 × 250 v2) (Illumina Inc., San Diego, CA, USA) at Microsynth AG (Balgach, Switzerland).

The DNA library for fungi was prepared in triplicate reactions in a two-step PCR approach using the 1389F and the ITS4ngsUni primer pair to target the full internal transcribed spacer region (ITS) (Tedersoo, Tooming-Klunderud and Anslan 2018). The replicated PCR products were pooled and then barcoded using the Barcoded Universal F/R Primers (Pacific Biosciences of California, Part number 100-466-100). We included a negative control (DNA was replaced by water) in all PCR runs. Further details on the primer, PCR reactions and cycling conditions are provided in Table S3 (Supporting Information). After a final quality check on agarose gels and quantification of the final DNA concentrations by fluorometry, the PCR products were pooled in equimolar amounts. PCR products after the first and second PCR and the final pooled library were purified with a magnetic bead solution (https://openwetware.org/wiki/SPRI_bead_mix).

Library preparation and sequencing was performed at the Functional Genomics Center Zurich (FGCZ, Zürich, Switzerland) by single-molecule, real-time sequencing technology using the Pacific Biosciences Sequel II System. The DNA Template Prep Kit 1.0 (Pacific Biosciences p/n 100-259-100) was used to produce the SMRT bell and the size and integrity of the amplicons assessed with a Bioanalyser 2100 12K DNA Chip assay (Agilent p/n 5067-1508). DNA (600 ng) was end-repaired using polishing enzymes. After exonuclease treatment, a blunt-end ligation reaction was conducted to create the SMRT bell template. The SMRT library was quality inspected and quantified on the Agilent Bioanalyser 12 Kb DNA Chip and a Qubit Fluorometer (Life Technologies), respectively. According to the manufacturer's instructions, a ready-to-sequence SMRTbell Polymerase Complex was created using the Sequel binding kit 3.0 (Pacific Biosciences p/n 101‐500‐400). The Pacific Biosciences Sequel instrument was programmed to sequence the library on 1 Sequel SMRT^®^ Cells 1M v3 (Pacific Biosciences p/n 101‐531‐000), taking one movie of 10 h/cell, using the Sequel Sequencing Kit 3.0 (Pacific Biosciences p/n 101‐597-900).

### Bioinformatics

Illumina reads were demultiplexed using mothur version 1.44.1 (Schloss *et al*. 2009). PacBio subreads were demultiplexed using the demultiplex barcodes application (minimum barcode score = 45) in the PacBio software smrt link (version 6.0.0.47841). Circular consensus sequencing (CCS) reads were generated from the demultiplexed subreads using the CCS application (minimum number of passes = 5, minimum predicted accuracy = 0.999) in the same PacBio software package. The remaining steps in the bioinformatics pipeline were performed at Scientific Computer Cluster Euler at ETH Zurich: The removal of phiX was confirmed in the Illumina data. The 16S rRNA MiSeq read ends were trimmed (Trim R1: 10, Trim R2: 15) before merging (min. overlap: 15, max. overlap: 300, max. mismatch density: 0.25), and primers were removed, allowing one mismatch with usearch v11.0.667 (Edgar 2013). For ITS PacBio data, primers were removed with usearch allowing two mismatches. The quality of the reads was further filtered with prinseq-lite 0.20.4 (Schmieder and Edwards 2011); for 16S rRNA MiSeq reads, amplicons with a size between 200 and 450 bp were selected and for ITS PacBio between 450 and 1200 bp (both data sets: GC range 30–70%, min. Q mean 20, low complexity filter dust with a threshold 30). Remaining sequences were denoised with usearch (Edgar 2016) and clustered into operational taxonomic units (OTUs; OTU with zero radius and abundance threshold of 8) at 97% sequence similarity with unoise3 as part of usearch v11.0.667. Singletons were removed (abundance threshold 2), and taxonomy was assigned with sintax (Edgar 2016) either with the silva v128 database (Quast *et al*. 2012) for the 16S rRNA gene region or with unite V7.2 (Quast *et al*. 2012) for the ITS gene region; the fungal annotation was confirmed with ITSx (Bengtsson‐Palme *et al*. 2013). Chimeric sequences were removed. The bioinformatics report file is available online as Supporting file 1 (Supporting Information).

## STATISTICAL ANALYSES

RStudio (RStudio Team 2016), a development environment for r (R Core Team 2019), was used to analyse the data; graphs were created in the R package ggplot2 (Wickham 2016) and forestplot (Gordon and Lumley 2019).

### Univariate data sets

Bayesian mixed models were fitted using Stan's probabilistic programming language (Carpenter *et al*. 2017) for full Bayesian inference through the R package brms (Bürkner 2017), version 2.10.0; data collected before setting up the experiment were analysed separately from the other data by including the farming system as fixed effect and the field plot ID nested in field block as a random effect in the model (see Figure S2, Supporting Information, for a field map). Data from T1 to T3 were analysed jointly with a separate model for each response variable using the main experimental factors (system, time and drought) with all two- and the three-way interactions as fixed effects; the random effects remained as described. To assess the effect of AMF abundance on plant-related data on T3 (grain and straw yields, total shoot biomass and plant height), we run linear regression models, including the plot ID in the random part. Because growth regulators were applied in the ConMin but not of the BioDyn system, we run these models separately for the two farming systems. A Gaussian error distribution was assumed for all models in this study except for weed coverage (continuous proportions; 0 < *x* < 1), where a beta regression model was fitted. For all models, the weakly informative default priors of brms were used. The posterior distribution was simulated using five chains and twenty thousand effective samples for each parameter. Sampling quality and model fit was assessed visually and numerically through the shinystan web interface (Gabry 2018). From the posterior distribution of the model parameters, median values and differences between selected treatment groups were calculated together with their 95% credible intervals (CrIs) using the 97.5 and 2.5% quantiles as the upper and lower limits. The results of the data analyses are mainly presented in graphical forms. Non-overlapping of an estimate with the credible interval of another estimate can be regarded as analogous to a significant difference in the classical frequentist framework (given flat/weakly informative priors). Throughout the manuscript, we mainly discuss differences between roof and control but show the values of the roof control in the central figures and tables.

### Sequencing data sets

Before downstream analysis, non-bacterial and non-fungal OTUs were removed from the data. This included the removal of archaeal sequences from the 16S rRNA sequences data, which we did not analyse further because of low sequences coverage (median: 69.5 sequences, range: 24–218 sequences). Following McMurdie and Holmes (2014), data were not rarefied. Using phyloseq (McMurdie and Holmes 2013), the Shannon index (‘H’; Shannon 1948) was calculated, from which the Shannon diversity [D = exp(H)] was obtained following Jost (2006). The data were then analysed in the Bayesian framework as described above. For the subsequent analysis, the data were filtered to remove rare OTUs (fewer than 20 reads occurring in fewer than 5% of the samples). After filtering, the data consisted of 793 737 bacterial sequences (range: 6287–26 700; median: 10 202) and 109 020 fungal sequences (range: 450–2837; median: 1491), which were clustered into 2763 unique bacterial OTUs and 357 unique fungal OTUs. Rarefaction curves for the filtered data are provided in Figure S3 (Supporting Information); details on the effect of filtering are provided in Table S5 (Supporting Information). If not explicitly mentioned, all following analyses were based on normalised (relative abundance) OTU tables. The effect of the experimental factors on the microbial community compositions was analysed by permutational multivariate analysis of variance (PERMANOVA) in vegan (Oksanen *et al*. 2019) based on the Bray–Curtis dissimilarity matrix. To identify differences between levels of significant treatment factors, multilevel-pairwise post-hoc tests with Bonferroni corrections for multiple testing were performed using the pairwiseAdonis package (Martinez Arbizu 2019). Considering that differences in group dispersion can affect the interpretation of PERMANOVA results, the homogeneity of multivariate dispersion in the treatment groups was explored applying the functions *vegan::betadisp* and *anova*. Finally, significant factors of the PERMANOVA were subjected as constraining terms in a distance-based redundancy analysis (db-RDA) using the *vegan*::*dbrda* function, restricting permutations on the field blocks. As before for the PERMANOVA test, we based our analysis on the Bray–Curtis dissimilarity matrix. To visualise environmental variables that correlate with the ordination projections, the respective vectors were plotted onto the db-RDA ordinations using *vegan*::*envfit* with linear combinations of scores, restricting the graphical presentation to those variables that exceeded an *a priori* threshold of *r*^2^ > 0.4. To detect bacterial and fungal OTUs explicitly associated with one of the two farming systems, the roof or the control subplots, we performed an indicator species analysis using the function *multipatt* within the indicspecies package (De Caceres and Legendre 2009). The filtered (fewer than 20 reads occurring in fewer than 5% of the samples were removed) OTU count tables were used as input and the association between bacterial or fungal OTUs with the experimental factors determined based on the point biserial correlation coefficient and 999 permutations. More specifically, we screened for indicator OTUs in the farming systems across the three sampling dates and drought treatments. We screened for indicator OTUs associated with either the control or the roof subplot separately within each sampling date within farming systems. By only showing OTUs with a point biserial correlation coefficient > 0.6 and a *P* < 0.01, we restrict the results to the indicator OTUs with closest association to the experimental factors.

## RESULTS

### Basic site description

Before starting the experiment, we assessed essential soil characteristics in areas close to the subplots to confirm and describe differences between the two farming systems. Compared with the ConMin system, the BioDyn system had higher soil pH, total soil carbon and nitrogen, along with a tendency for higher soil water holding capacity and slightly lower bulk density, whereas the soils in the two different farming systems were comparable in soil phosphates and soil phosphorus (Table S4, Supporting Information).

### Roof and farming system effects on soil water content

**Figure 1.**
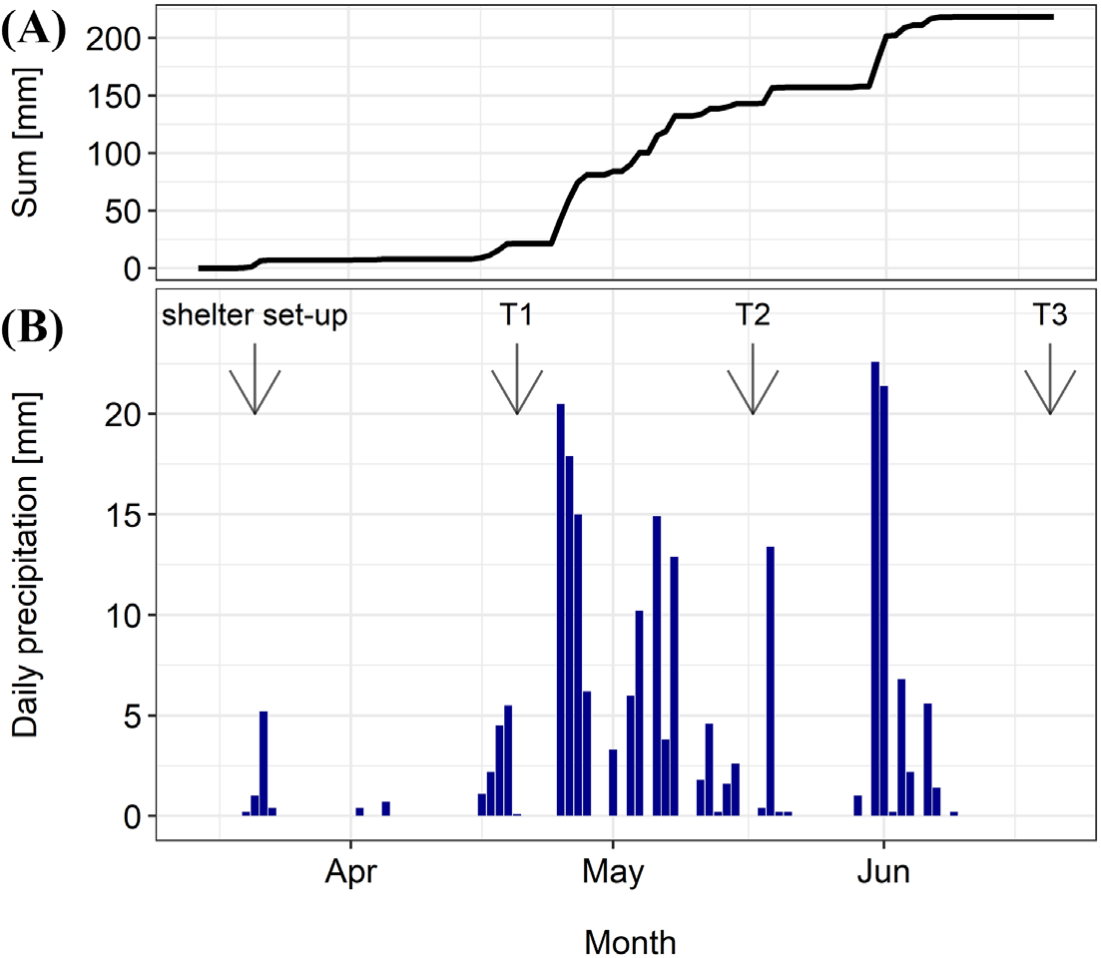
Precipitation levels (mm) on the study site. **(A)** Cumulative precipitation; **(B)** daily precipitation sum; arrows show start of the experiment (rainout shelter set-up) and the three sampling dates conducted 4 (T1), 8 (T2) and 13 (T3) weeks after rainout shelter set-up.

**Figure 2.**
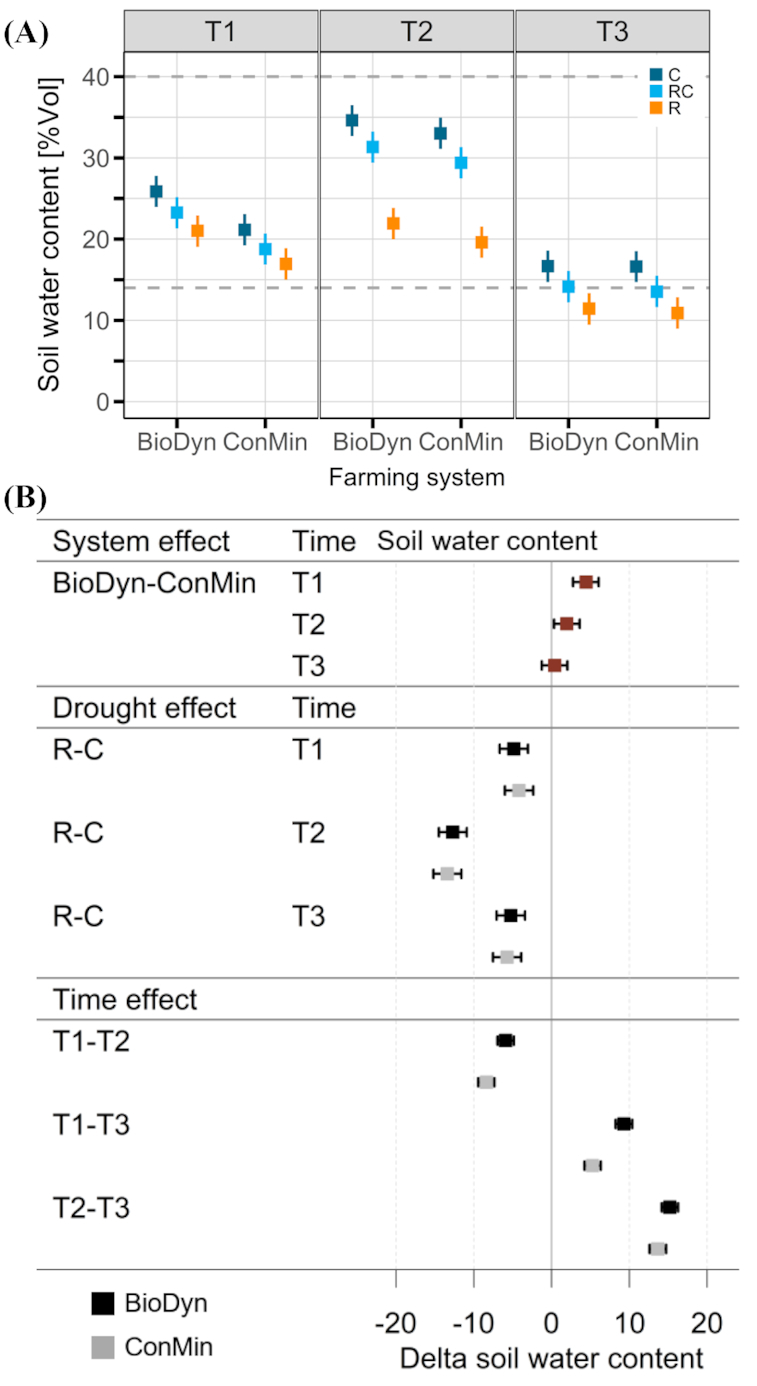
**(A)** Volumetric soil water content; dashed lines: estimated wilting point (bottom), field capacity (top); **(B)** differences in soil water content (in percentage points); system and time effect are given as averages over the drought treatments. All data are presented as medians of the posterior distribution with 95% credible intervals (CrIs). Factor system: biodynamic farming system (BioDyn), conventional farming system with pure mineral fertilisation (ConMin); factor drought: control (C, no shelter), rainout shelter control (RC), rainout shelter (R); factor time: 4 (T1), 8 (T2) and 13 (T3) weeks after rainout shelter set-up.

The precipitation sum and daily precipitation levels are shown in Fig. 1. The roof, on average, excluded 65% of the incoming precipitation (Kundel *et al*. 2018) and, in comparison with the roof control and the control, successfully reduced soil water content on all three sampling dates (Fig. 2A). On T2, we recorded the highest soil water content since the start of the experiment along with the most pronounced differences between roof and control subplots (Fig. 2B); however, the soil water content in the roof subplots was still relatively high (Fig. 2A). On T3, the soil water content dropped below the estimated wilting point in the roof, but not in the control subplots (Fig. 2A). On all sampling dates, the observed differences in soil water content between the roof and the control subplots were similar in the two farming systems. When averaged over the drought treatments, soil water content was higher in the BioDyn system compared with the ConMin system on T1 and T2 but not on T3 (Fig. 2B).

### Basal respiration and microbial biomass

**Figure 3.**
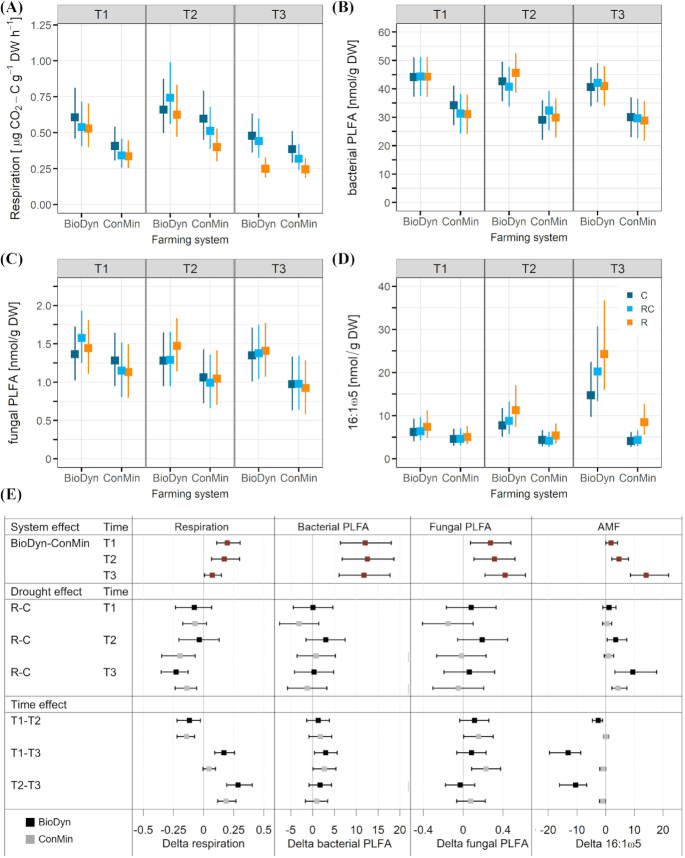
**(A)** Soil basal respiration; **(B)** bacterial PLFA marker abundance; **(C)** fungal PLFA marker abundance; **(D)** arbuscular mycorrhizal fungi (AMF) (16:1ω5) marker abundance; **(E)**differences between selected treatments for the response variables shown in A–D with system and time effects given as averages over the drought treatments. All data are medians of the posterior distribution with 95% credible intervals (CrIs). Factor system: biodynamic farming system (BioDyn), conventional farming system with pure mineral fertilisation (ConMin); factor drought: control (C, no shelter), rainout shelter control (RC), rainout shelter (R); factor time: 4 (T1), 8 (T2) and 13 (T3) weeks after rainout shelter set-up.

The soil basal respiration, which was used as an indicator of microbial activity, was affected by the farming system, sampling date and drought treatment (Fig. 3A). On all sampling dates, the basal respiration rates, averaged over the drought treatments, were higher in the BioDyn compared with the ConMin farming system. The basal respiration was highest on the wettest sampling date (T2) and lowest on the driest sampling date (T3).

In both farming systems, bacterial and fungal PLFA marker abundance remained mostly unaffected by the sampling dates and the drought treatments (Fig. 3B and C). However, bacterial and fungal abundance was affected by the farming system, with higher abundance in the BioDyn compared with the ConMin farming system (Fig. 3B and C). We found effects of the farming system, sampling date and drought treatment on AMF abundance (NLFA 16:1ω5) (Fig. 3D): In the BioDyn farming system, AMF abundance increased from T1 to T3 in the roof subplots in particular, whereas in the ConMin system, the marker was generally lower than in the BioDyn system and increased only after T2 under the roof. All treatment comparisons on basal respiration and microbial abundance that are mentioned in the text are highlighted in Fig. 3E.

### Alpha- and beta diversity of bacterial and fungal communities

**Figure 4.**
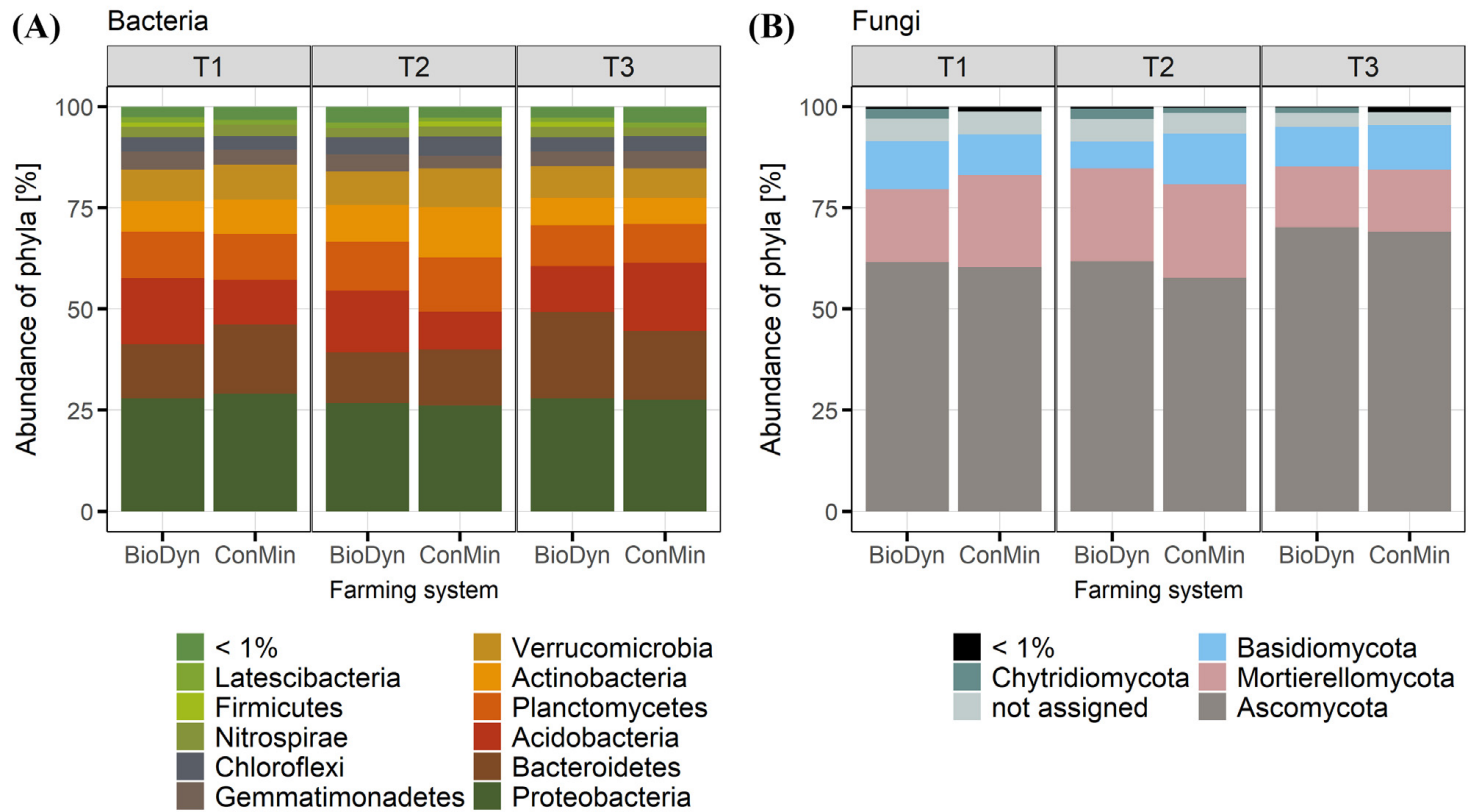
Relative abundance of operational taxonomic units agglomerated on the phylum level for **(A)** bacteria and **(B)** fungi. Bars show mean relative abundance across the levels of the drought treatment and the field plots. Factor system: biodynamic farming system (BioDyn), conventional farming system with pure mineral fertilisation (ConMin); factor time: 4 (T1), 8 (T2) and 13 (T3) weeks after rainout shelter set-up.

To characterise the fungal and bacterial communities in more detail, we used amplicon-based sequencing. In total, we found 11 bacterial phyla of which the 5 most abundant ones were classified as *Proteobacteria*, *Bacteroidetes*, *Acidobacteria, Planctomycetes* and *Actinobacteria* (Fig. 4A). Fungal communities were composed of *Ascomycota*, *Mortierellomycota*, *Basidiomycota* and *Chytridiomycota* (Fig. 4B).

**Figure 5.**
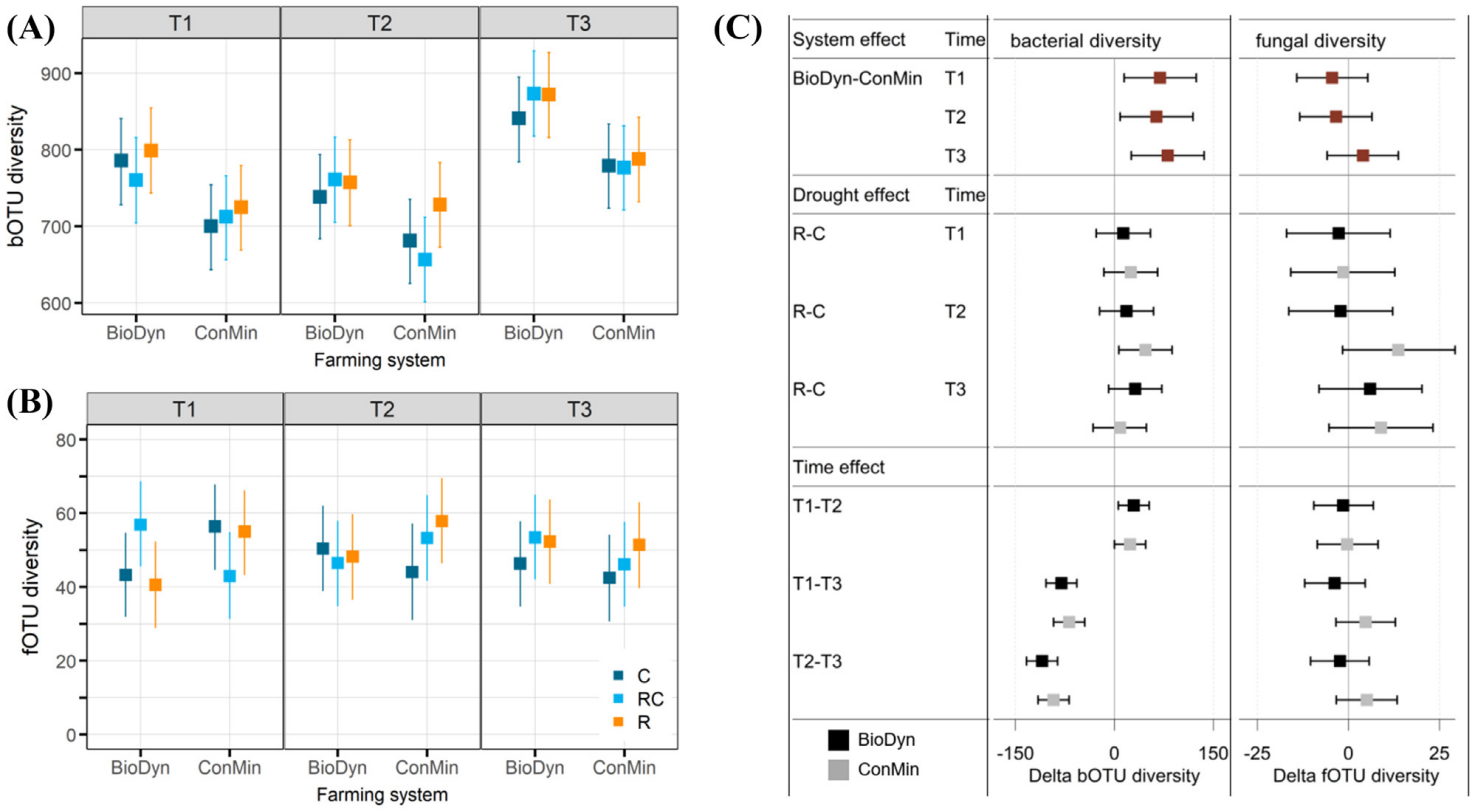
Shannon diversity of **(A)** bacterial operational taxonomic units (bOTUs) and **(B)** fungal operational taxonomic units (fOTU); **(C)** differences between selected treatments for the response variables shown in A and B with system and time effects given as averages over the drought treatments. All data are medians of the posterior distribution with 95% credible intervals (CrIs). Factor system: biodynamic farming system (BioDyn), conventional farming system with pure mineral fertilisation (ConMin); factor drought: control (C, no shelter), rainout shelter control (RC), rainout shelter (R); factor time: 4 (T1), 8 (T2) and 13 (T3) weeks after rainout shelter set-up.

**Figure 6.**
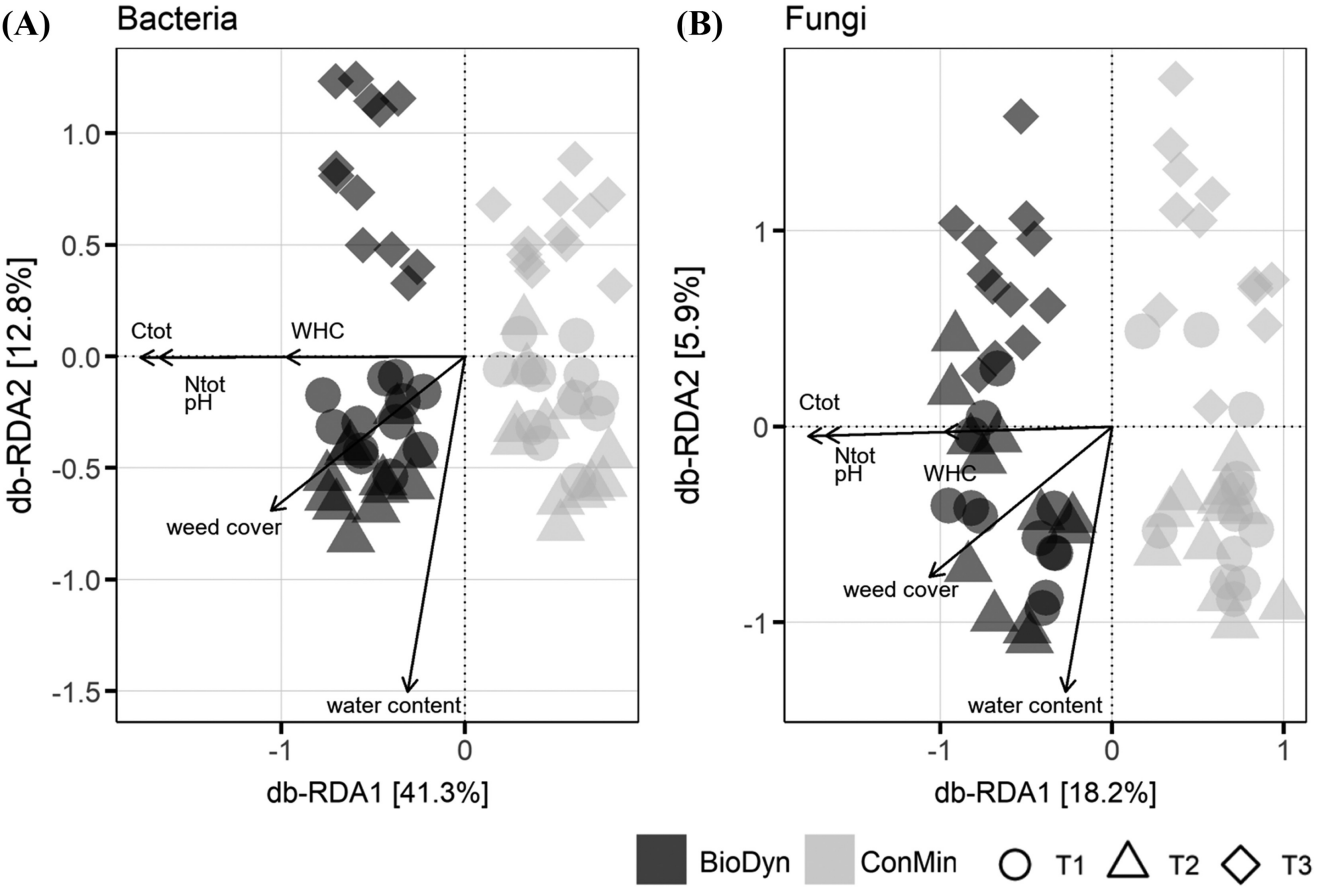
Distance-based redundancy analysis (db-RDA) constrained for farming system and sampling date and conditioned for field blocks for**(A)** bacteria and **(B)** fungi. Arrows show correlations between soil and site properties and the ordination scores (for correlations with *r*^2^ ≥ 0.4 and *P* < 0.01); arrow lengths are scaled according to the correlation strength (*r*^2^). Factor system: biodynamic farming system (BioDyn), conventional farming system with pure mineral fertilisation (ConMin); factor time: 4 (T1), 8 (T2) and 13 (T3) weeks after rainout shelter set-up. C_tot_: total soil carbon (in the field C_tot_ is all organic carbon); N_tot_: total soil nitrogen; WHC: water holding capacity; water content: volumetric soil water content.

The bacterial diversity (Shannon diversity) was highest on the driest sampling date (T3) and lowest on the wettest (T2) sampling date, but did not differ in response to the experimental drought within sampling dates in the two farming systems (Fig. 5A). Averaged over the drought treatments, the Shannon diversity of bacteria was higher in the BioDyn compared with the ConMin system on all sampling dates (Fig. 5A). Fungal Shannon diversity was mostly unaffected by the experimental factors (Fig. 5B). Differences in bacterial and fungal Shannon diversity in response to the experimental treatments are highlighted in Fig. 5C. The community composition (beta diversity) of bacteria and fungi was affected by the farming systems and the sampling dates but not by the experimental drought (PERMANOVA test; Table 1). The farming system effect (first axis) and effect of the sampling date (second axis) became evident in a constrained ordination (db-RDA; Fig. 6) and confirmed results of the multivariate post-hoc test (Table S6, Supporting Information), namely that microbial communities on T1 and T2 were more similar to each other than to communities on T3. Of the 12 soil physical and chemical parameters tested, 6 correlated strongly (*r*^2^ ≥ 0.4 and *P* < 0.001) with the ordination projections for bacteria and fungi (Table S7, Supporting Information). These were volumetric soil water content, soil water holding capacity, soil pH, total soil carbon and nitrogen content, and the soil surface covered by weeds (Fig. 6). Differences in multivariate spread can confound the results of a PERMANOVA. In bacterial communities, the multivariate spread differed between the two farming systems (*F* = 18.58; *P* < 0.001) in the sense that the bacterial community was slightly more heterogeneous in the BioDyn system compared with the ConMin system. Considering the apparent location effect (biological dissimilarity; Fig. 6A), this result indicates that the farming system effect is driven by both actual biological dissimilarities and differences in multivariate dispersion (variance). No differences in multivariate spread was found in fungal communities.

**Table 1.** Results of PERMANOVA assessing the effects of farming system (system), sampling date (time), rain manipulation (drought) and two-way interactions including drought on **(A)** bacterial and **(B)** fungal community composition based on the amplicon-based sequencing data and Bray–Curtis dissimilarity matrix. Factor drought: control without shelter, rainout shelter control, rainout shelter; factor time: 4, 8 and 13 weeks after rainout shelter set-up; factor system: biodynamic farming system and conventional farming system with pure mineral fertilisation.

Source of variation	Degrees of freedom	Sum of squares	*r* ^2^	Pseudo*F*-value	Pr (>*F*)
**(A)**Bacterial communities					
Drought	2	0.095	0.020	1.000	0.362
Time	2	0.443	0.094	4.675	0.001
System	1	1.121	0.238	23.693	0.001
Drought × system	2	0.073	0.016	0.776	0.788
Drought × time	4	0.143	0.030	0.755	0.898
Residuals	60	2.840	0.602	—	—
Total	71	4.715	1.000	—	—
**(B)** Fungal communities					
Drought	2	0.265	0.026	1.073	0.311
Time	2	0.529	0.052	2.143	0.001
System	1	1.367	0.135	11.077	0.001
Drought × system	4	0.407	0.040	0.824	0.897
Drought × time	2	0.188	0.019	0.762	0.917
Residuals	60	7.405	0.729	—	—
Total	71	10.161	1.000	—	—

### Indicator species analysis

**Figure 7.**
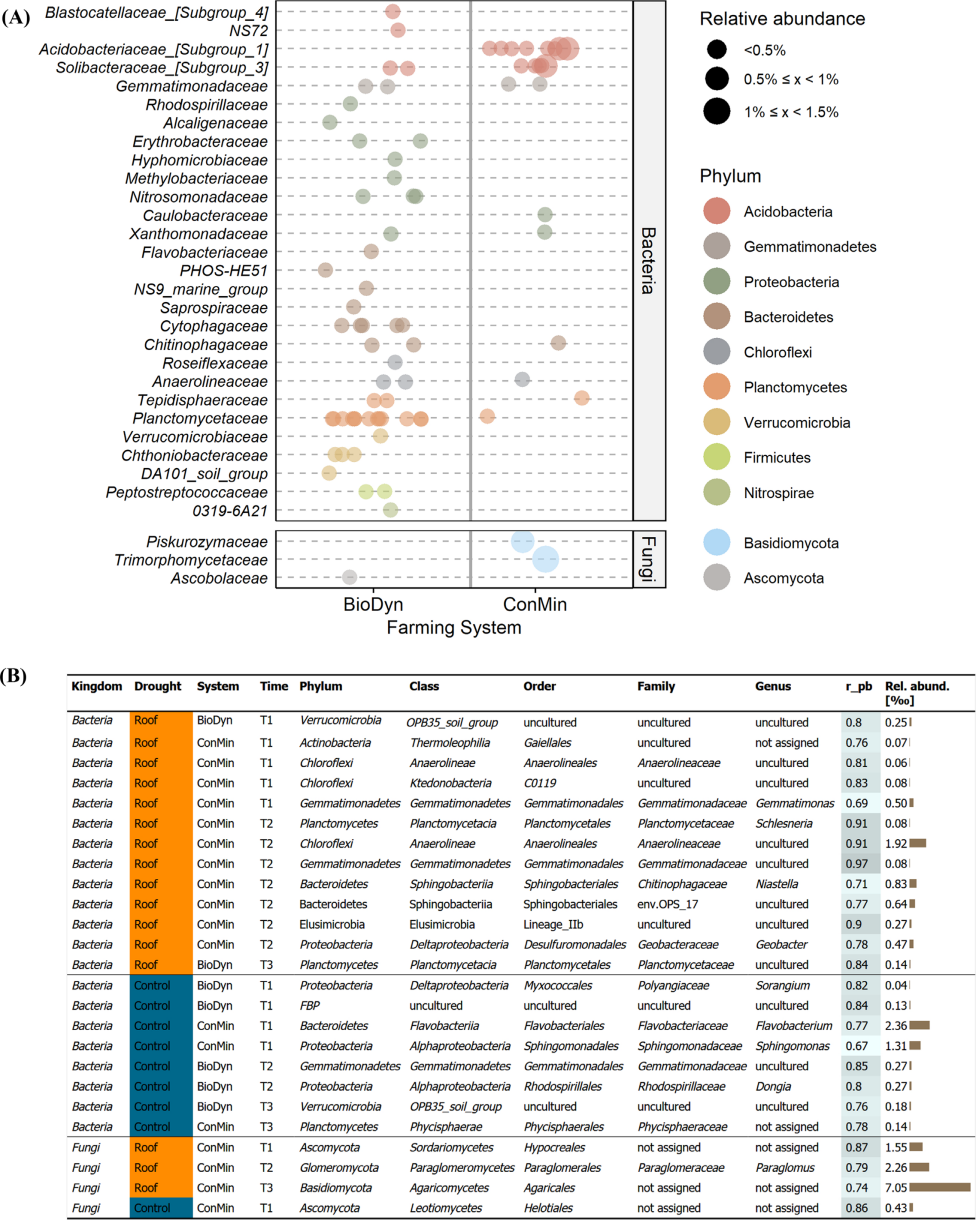
**(A)** Bacterial and fungal indicator species identified in the biodynamic (BioDyn) and conventional (ConMin) farming systems. Taxa are agglomerated on the genus level and coloured according to the annotation on the phylum level. Only taxa with a point biserial correlation coefficient > 0.6 and a *P*-value < 0.01 and annotation on the family level are shown. Dot sizes show the relative abundance of the indicator taxa (relative to the total bacterial or fungal sequence counts in the given farming system); **(B)** bacterial and fungal indicator species identified in the rainout shelter (R) or in the control (C) subplots. The analysis was performed separately for the three sampling dates (T1, T2 and T3), separately within the biodynamic (BioDyn) and conventional (ConMin) farming systems. The relative abundance is expressed in per mill as abundance of the indicator taxa relative to the total bacterial or fungal sequence counts in the given farming system and the given sampling date. Only taxa with a point biserial correlation coefficient (r_pb) > 0.6 and a *P*-value < 0.01 are shown.

To identify taxa explicitly associated with the farming system or the drought treatment, we performed an indicator species analysis. Fifty-three bacterial OTUs were identified as indicators for the BioDyn, and 21 for the ConMin system. In the BioDyn system, the identified indicators belonged to 9 phyla and 26 families of which the majority of indicators being found in the *Planctomycetaceae* (13 indicators) and *Cytophagaceae* (5 indicators). Bacterial indicators detected in the ConMin system belonged to 6 phyla and 9 families, the highest number being found within the *Acidobacteriaceae [Subgroup_1]* (8 indicators) and *Solibacteraceae [Subgroup_3]* (5 indicators). We detected five fungal indicators in the BioDyn and three in the ConMin system. A graphical summary on the bacterial and fungal indicator detected in the two farming systems is provided in Fig. 7A and more information in Tables S8–S10 (Supporting Information).

We further screened for taxa indicating a response to the drought treatment (roof and control). In the BioDyn system, there was one bacterial indicator on T1 and one on T3 that was specifically associated with the roof subplot (Fig. 7B). In the ConMin system, 11 indicators were identified for the roof subplot, of which 7 were detected on T2. The most abundant of all these indicators was a taxon within the family *Anaerolineaceae* belonging to the phylum *Chloroflexi* (Fig. 7B). In both farming systems, we found bacterial indicator for the control subplots, yet they were widely distributed across different phyla, and no clear clustering was apparent. We did not detect fungal indicator for the roof or the control treatments, in the BioDyn farming system on none of the sampling dates. In the ConMin system, we detected three indicators for the roof and one for the control subplots (Fig. 7B).

### Wheat growth, yields and weed cover

**Figure 8.**
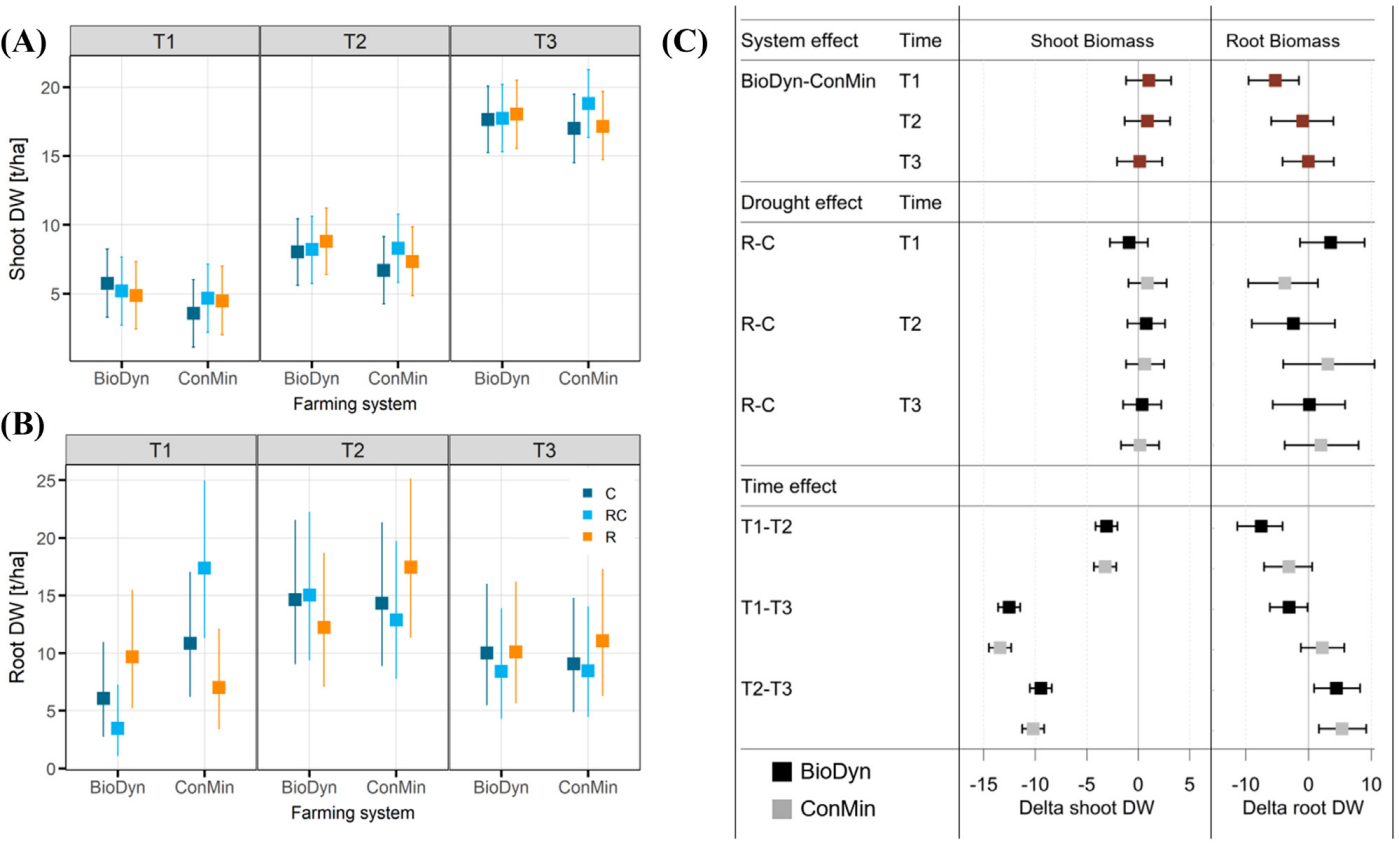
**(A)** Total shoot biomass (grain and straw) [t/ha]; **(B)** total root biomass [t/ha] in the top 20 cm of soil; **(C)** differences between selected treatments for the response variables shown in A and B [t/ha], with system and time effect given as averages over the drought treatments. All data are medians of the posterior distribution with 95% credible intervals (CrIs). Factor system: biodynamic farming system (BioDyn), conventional farming system with pure mineral fertilisation (ConMin); factor drought: control (C, no shelter), rainout shelter control (RC), rainout shelter (R); factor time: 4 (T1), 8 (T2) and 13 (T3) weeks after rainout shelter set-up.

To explore the potential consequences of our experimental treatments on provisioning ecosystem services, we measured a range of plant-related properties. Total shoot dry weight increased over time, but differences between the drought treatments or farming system were not detected (Fig. 8A), even though the plants in the BioDyn compared with the ConMin system were taller on all sampling dates (Figure S4, Supporting Information). Root biomass in the top 20 cm of the soil profile increased between T1 and T2 and decreased between T2 and T3, but showed no apparent response to the drought treatment (Fig. 8B). Farming system effects on root biomass production were found only on T1; here root biomass was lower in the BioDyn compared with the ConMin system. The mentioned treatment comparisons for the shoot and root biomass are highlighted in Fig. 8C. Straw and grain yields measured on T3 did not differ between the drought treatments (Table 2). There was no apparent difference in straw and grain yields between the two farming systems, only a trend for higher straw yields (+10%, 95% CrIs: −6%, +28%) but lower grain yields (−13%, 95% CrIs: −28%, 4%) in the BioDyn compared with the ConMin farming system. Weed cover was higher in the BioDyn compared with the ConMin on T1 and T2, but not on T3. There was no effect of the experimental drought on weed cover in none of the two farming systems (Figure S5, Supporting Information).

**Table 2.** Final **(A)** grain and **(B)** straw yields at the last sampling date (T3). Data are presented as median of the posterior distribution with 95% credible intervals (95% CrIs). Factor system: biodynamic farming system (BioDyn), conventional farming system with pure mineral fertilisation (ConMin); factor drought: control (C, no shelter), rainout shelter control (RC), rainout shelter (R).

**(A)**Grain yields [t/ha]				**(B)** Straw yields [t/ha]			
System	Drought	Median	95% CrI	System	Drought	Median	95% CrI
BioDyn	C	6.08	4.48, 7.69	BioDyn	C	11.59	9.41, 13.78
	R	6.20	4.57, 7.83		R	11.84	9.68, 14.06
	RC	6.07	4.42, 7.72		RC	11.68	9.54, 13.87
ConMin	C	6.74	5.14, 8.41	ConMin	C	10.27	8.18, 12.53
	R	6.83	5.22, 8.51		R	10.34	8.18, 12.64
	RC	7.48	5.87, 9.13		RC	11.35	9.20, 13.57

### Effects of AMF abundance on plant-related data

To determine the potential effects of AMF abundance on plant-associated parameters, we performed linear regressions, separately for each farming system. We found no relationships between AMF abundances and plant-related properties (grain yield, straw yield, total shoot biomass or plant height) in either the BioDyn or the ConMin system (Table S11, Supporting Information).

## DISCUSSION

According to global climate models, summer months in central and southern European countries will be characterised by increasingly frequent and severe droughts (Pachauri *et al*. 2014) with predicted negative consequences for agricultural production across Europe (Webber *et al*. 2018) and worldwide (Daryanto, Wang and Jacinthe 2017). The enhanced resistance of soils mediated through high levels of SOC and improved soil biological quality achieved through extensive management has been proposed to counteract the adverse effects of climate change (Goh 2011). Here, we investigated the influence of organic (biodynamic) and conventional farming systems on the response of microbial communities, wheat growth and yields to short-term drought periods under field conditions, a subject rarely investigated before.

### Agricultural production under future precipitation levels may require specific actions beyond organic farming

We hypothesised that the soils under long-term biodynamic management generally contain more water than soils under conventional management. This assumption was based on the high SOC levels in organically managed soils (Gattinger *et al*. 2012) and the positive effects of SOC on plant available water content (Huntington 2006). Indeed, we found enhanced SOC contents in the biodynamic compared with the conventional farming system along with higher soil water content at times when soil water was not limiting (T1 and T2). However, in both farming systems, soil water content dropped to similarly low levels on the driest sampling date (T3), and the relative reduction in soil water content (roof vs control) was comparable for the two farming systems. The ultimate effect of SOC on soil water content is complex and depends not only on the quantity of SOC but also its physical and chemical properties and the texture of a given soil (Rawls *et al*. 2003; Huntington 2006). In general, however, the influence of SOC on soil water content seems to decrease with decreasing water potential (Rawls *et al*. 2003; Huntington 2006; Minasny and McBratney 2018), which is also reflected in our findings. Our results indicate that, in addition to careful carbon management, other measures specifically tailored to reduce soil evaporation, transpiration and excessive evapotranspiration may be needed to protect soils from drying. Such measures may include a surface cover with plant residues or living mulches, agroforestry or intercropping in combination with the cultivation of water-efficient crops (Lal and Francaviglia 2019).

### Short-term drought impairs basal respiration and promotes the abundance of arbuscular mycorrhizal fungi

Microbial basal respiration depends strongly on soil water content because of the water's vital role in substrate diffusion, which in turn directly impacts on the availability of nutrients to microbes (Manzoni *et al*. 2012). Therefore, the higher basal respiration rates on T2 compared with T1 indicate that soil moisture did not decrease to levels that exposed microorganisms to a stressful situation. Between T2 and T3, however, soil moisture dropped to values around the wilting point and, as expected, respiration rates decreased; however, the decline in respiration did not differ between the farming systems. The soil water content was critically low on T3, and, contrary to our assumption, did no longer differ between the two farming systems, hence substrate diffusion likely restricted microbial respiration in both systems equally. A higher respiration in the biodynamic compared with the conventional farming system was only found under optimal water content, thus, our findings emphasise the need to prevent soils from drying out to benefit from the positive effects of biodynamic farming on basal respiration.

Bacterial and fungal abundances as assessed by PLFA remained largely stable throughout the experiment and were not affected by the drought treatments. Severe drought may lead to a decline in microbial biomass (Homyak *et al*. 2017; Ren *et al*. 2018), e.g. due to enhanced microbial mortality. However, as mentioned, in our study, soil moisture levels dropped to a critical threshold (wilting point) only after the second sampling. To adapt to moderate or short-term drought, microbes can form spores or resting structures (Sharma and Gobi 2016; Schimel 2018) without suffering severe declines in biomass. Moreover, the adverse effects of drought on sensitive predators of microbes (e.g. bacterivorous nematodes: Kardol *et al*. 2010; Landesman, Treonis and Dighton 2011; or protists: Geisen *et al*. 2014) can reduce predation pressure (Schimel 2018) and may counteract the direct adverse effects of drought on microbial biomass.

The abundance of AMF increased under the experimental drought, in line with previous findings (Augé 2001; Karlowsky *et al*. 2018; Mackie *et al*. 2019). AMF associations can play a crucial role in the plants’ access to water (Khalvati *et al*. 2005), and plants can promote the association with the fungus via their carbon allocation (Simard and Austin 2010; Pagano 2014). The relative increase in AMF abundance (roof compared with control) was comparable for the two farming systems, yet, only the biodynamic farming system promoted AMF abundance in addition to the experimental drought. Differences in AMF abundances in the two farming systems could be related to the quantity and quality of the applied fertilisers (Mäder *et al*. 2000; Oehl *et al*. 2004) or the higher weed prevalence in the biodynamic compared with the conventional farming system, given that weeds can act as additional hosts. The additive, positive effects of the experimental drought and the biodynamic farming system resulted in almost three times higher AMF abundance in the roof subplots of the biodynamic compared with those of the conventional farming system at times of the most severe drought (T3).

Interestingly, AMF abundance was not related to the crop's plant performance under drought. Several factors can explain this observation: (i) the duration of the drought may have been too short such that the increased AMF abundance could have been reflected in the plant-related data or, (ii) the effect of the increased AMF abundance was reflected in plant-related parameters other than biomass and yield, e.g. changes on the physiological or cellular level. Finally, the reason that the plants did not respond to the experimental drought may be precisely the increased AMF abundance, in the sense that the increased AMF abundance helped to prevent a possible negative drought effect on the plant. Further analyses using more specific methods, may shed light onto the role of AMF in buffering yield losses during drought periods in differently managed farming systems. Such methods may include amplicon-based sequencing approaches (e.g. Schlaeppi *et al*. 2016; Symanczik *et al*. 2017) in bulk soil but also in root samples where the actual symbiotic relationship between the plants and fungi is established. Such data will help to illuminate the effects of the farming system and drought on the proportion of AMF that directly interact with crops and may better depict the consequences for plant growth and final yield.

### Microbial diversity and community composition are stable under simulated drought

Bacterial and fungal Shannon diversity (alpha diversity) as assessed from amplicon-based sequencing was not affected by the experimental drought on any of the sampling dates. However, independent of farming system or experimental drought, across the season, bacterial diversity was highest on the driest sampling date and lowest on the wettest sampling date. In contrast, no such relationship with soil water over time was observed for fungi. Carson *et al*. (2010) demonstrated that low pore connectivity caused by low water content promoted Shannon diversity of soil bacteria. These authors argued that dry pore spaces create the isolated habitats and niches that shelter less competitive bacteria. Seaton *et al*. (2020) further showed that fungi are less constrained by their physical environment compared with bacteria, which might be related to the hyphal system that allows them to bridge dry pore spaces (Tecon and Or 2017).

Like alpha diversity, the composition of bacterial and fungal communities (beta diversity) was not affected by changes in soil water content as created by the roof. However, the farming system was a relevant factor shaping microbial community properties, which is in line with earlier findings (Hartmann *et al*. 2015; Bonanomi *et al*. 2016; Lori *et al*. 2017; Lupatini *et al*. 2017, 2019; Hartman *et al*. 2018; Harkes *et al*. 2019). Moreover, changes in soil water content across the season were among the most relevant drivers of the composition of microbial communities. Our findings indicate that the alpha diversity of bacteria and community composition of both bacteria and fungi react to substantial changes in soil water content; in our study, such changes were created only across the growing season. However, shifts in soil water contents over time co-occurred with changes in other environmental variables, including soil and air temperature and the plants’ growth stage. Given this, our data do not allow us to directly relate the observed patterns in microbial alpha and beta diversities across the season to changes in soil water contents. If we could maintain substantial differences in soil water contents between rainout shelters and control treatments at times of severe drought, we could compare community properties on the same sampling date and, therefore, separate changes in soil water from other potentially influencing factors. This separation could be achieved by irrigating the control subplots in times with overall low precipitation levels (Beier *et al*. 2012). We regarded irrigation as too artificial and, for practical reasons, not feasible in our study. Nonetheless, watering the control subplots should be considered in future studies with passive rainout shelters during times of drought and when the soil water content is at risk of falling below a critical threshold.

### Indicator species analyses

Among the most prominent families within the identified bacterial indicators in the biodynamic farming system were the *Planctomycetaceae* within the phylum *Planctomycetes*. This phylum has previously been found to be characteristic for organic farming systems (Lupatini *et al*. 2017), and its importance for the decomposition of soil organic carbon (Wang *et al*. 2015) may explain the prominent role in the organically fertilised soils in our study. In the biodynamic farming system, the genus *Flavobacterium* had the highest sequence abundance of all indicator OTUs. This bacterium has previously been found to be prominent in organic farming systems (Bonanomi *et al*. 2016; Armalytė *et al*. 2019) and organic-rich soils in general (Bernardet and Bowman 2006). Finally, the *Peptostreptococcaceae*, a genus within the *Firmicutes* appeared specifically in the organically managed system, in line with earlier reports in which *Firmicutes* were found to be associated with organic farming systems (Hartmann *et al*. 2015; Bonanomi *et al*. 2016; Hartman *et al*. 2018; Lori *et al*. 2018). In the ConMin system, most indicator OTUs were assigned to the family *Acidobacteriaceae* (Subgroup 1) and *Solibacteraceae* (Subgroup 3), which both belong to the phylum *Acidobacteria*. Members of *Acidobacteria* seem to be predominant in environments with moderately acidic pH conditions (Sait, Davis and Janssen 2006); however, our knowledge of the ecological role of the numerous subgroups within the *Acidobacteria* is still a field of current research (Kielak *et al*. 2016). The *Glomeromycota* did not appear on the list of fungal indicators, as they are only represented by very few sequences in the fungal data set (152 total sequences in the filtered data). More specific investigations on AMF may be required with primers designed to target this specific group (e.g. Schlaeppi *et al*. 2016). Only very few bacterial and fungal OTUs were associated with the samples from the drought subplots, and only a few of these drought indicator OTUs could be assigned to genus level, making it hard to extract information on their ecology or habitat preferences.

### Crop biomass and final crop yields are stable under short-term droughts

Grain yields are usually around 20% lower in organic compared with conventional farming (Mäder *et al*. 2002). In line with this, grain yields in our study were around 13% lower in the biodynamically managed fields; however, the uncertainty around this result was considerable, likely because of the relatively small size of the sampling area (0.1 m^2^). Yield levels in the current study are also higher as usual in the DOK trial (Mäder *et al*. 2002; Mayer *et al*. 2015). Although losses from threshing, separating and cleaning with modern machinery are nowadays relatively small, they might exceed those of the completely manual harvest in our current study. Furthermore, we removed neighbouring plants at each sampling date, which probably reduced competition and increased final yields. The nitrogen-fixing properties of soybean, the preceding crop, could also have promoted overall yield levels. However, we were interested in examining the relative differences between drought treatment levels, and the higher yield levels are not particularly relevant to our study. For more practice-oriented grain and straw yields from the DOK trial, we refer to earlier studies (Mäder *et al*. 2002; Mayer *et al*. 2015). Contrary to expectation, the simulated drought had no direct influence on the measured plant parameters (root and shoot biomass, straw and grain yields) in any of the farming systems, possibly because of the overall short duration of the simulated drought or the mentioned increased AMF abundance in the roof subplots.

### Implications and outlook

Understanding the functioning, potential and limitations of farming systems under the projected rainfall reductions is essential to adapt agricultural strategies and guide policy intervention. Drought-induced effects in the current experiment were small, hindering our ability to study the interactive effects of farming systems and drought. Still, we observed patterns with potential implications for successful crop production in a changing climate.

Overall, our data suggest that organic (biodynamic) agriculture enhances the soil's water storage capacity. Current climate models predict drought phases in summer to be preceded by heavy precipitation in spring. A high water storage capacity will be essential to replenish soil water reservoirs in spring and prevent surface run-off (Minasny and McBratney 2018). Having said this, the limited ability of SOC to enhance soil water contents under dry conditions should not diminish the importance of careful carbon management but rather encourage the implementation of additional strategies tailored to maintain soil water content under dry conditions. The interactions between farming systems and drought on microbial communities should be investigated under more substantial, extended and repeated droughts. The potential of AMF to help plants survive droughts can be further investigated in root samples from managed soils using more targeted methods. Such studies may contribute to the development of agricultural systems that remain productive even under predicted reduced rainfall.

## ACKNOWLEDGMENTS

We thank Sebastian Grau, Anton Kuhn, Jennifer Meier, Svenja Meyer, Adolphe Munyangabe, Frédéric Perrochet, Moritz Sauter and Marika Truu, for help with field and/or lab work. We further thank Fränzi Korner-Nievergelt and Pius Korner from oikostat.ch for their support with Bayesian data analysis, Jean-Claude Walser for help with bioinformatics, Jordi Moya Laraño for discussion of different statistical approaches, Mark van Kleunen for support with planning the experimental set-up and providing measuring devices, Emily Wheeler for proofreading of the first draft of the manuscript and the two anonymous reviewers for their valuable inputs during the revision process. DK further acknowledges support from the International Max Planck Research School for Organismal Biology.

## DATA AVAILABILITY

The sequencing data underlying this article are available in the NCBI Sequence Read Archive (SRA) database at https://www.ncbi.nlm.nih.gov/bioproject and can be accessed with the study accession number BioProject ID PRJNA641521.

## Supplementary Material

fiaa205_Supplemental_Files
