# Supplementary material for: Effects of simulated drought on biological soil quality, microbial diversity and yields under long-term conventional and organic agriculture
Source: FEMS Microbiol Ecol. 2020 Oct 5;96(12):fiaa205. doi: 10.1093/femsec/fiaa205 (PMC7705324; doi:10.1093/femsec/fiaa205)
Supplement: fiaa205_Supplemental_Files [file fiaa205_supplemental_files.zip › 20200920_Supplementary_RevisedVersion.docx]

# Supplementary material to: Effects of simulated drought on biological soil quality, microbial diversity and yields under long-term conventional and organic agriculture

Dominika Kundel^1,2*^, Natacha Bodenhausen^1^, Helene Bracht Jørgensen^3^, Jaak Truu^4^, Klaus Birkhofer^5^, Katarina Hedlund^3^, Paul Mäder^1^, Andreas Fliessbach^1^

*^1^Department of Soil Sciences, Research Institute of Organic Agriculture (FiBL), Frick, Switzerland*

*^2^Ecology, Department of Biology, University of Konstanz, Konstanz, Germany*

*^3^Department of Biology, Lund University, Lund, Sweden*

*^4^Institute of Molecular and Cell Biology, University of Tartu, Tartu, Estonia*

*^5^Department of Ecology, Brandenburg University of Technology, Cottbus, Germany*

*Corresponding author: Dominika Kundel, Research Institute of Organic Agriculture (FiBL), Ackerstrasse 113, CH-5070 Frick, phone: 0041 62 865 72 02, email: dominika.kundel@fibl.org

## Supplementary Tables

Table S1. Management operations performed in the winter wheat plots of the biodynamic (BioDyn) and the conventional farming system with pure mineral fertilisation (ConMin) during the 2016/2017 growing season.

| **Date** | **Farming system** | | **Activity** | **Details** | **Quantity** | | **Unit** |
| --- | --- | --- | --- | --- | --- | --- | --- |
|  |  |  |  |  | BioDyn | ConMin |  |
| **Year 2016** | | | | | | | |
| Oct., 04 | BioDyn | ConMin | Stubble working | spring tooth harrow,  10 cm deep | 1 | 1 | Passage |
| Oct., 11 | BioDyn | ConMin | Ploughing | --- | 1 | 1 | Passage |
| Oct., 11 | BioDyn | ConMin | Rolling | --- | 1 | 1 | Passage |
| Oct., 12 | BioDyn | --- | Manure and compost | --- | 70 | --- | dt/ha |
| Oct., 13 | --- | ConMin | Fertilisation | Triplesuperphosphat | --- | 30 | Kg P/ha |
| Oct., 13 | --- | ConMin | Fertilisation | Kali 60% | --- | 100 | Kg K/ha |
| Oct., 13 | BioDyn | ConMin | Seedbed preparation | spring tooth harrow,  10 cm deep | 2 | 2 | Passages |
| Oct., 14 | BioDyn | ConMin | Sowing | ConMin: seeds dressed (Celest Trio; 200ml/100kg) | 415 | 415 | grains/m^2^ |
| **Year 2017** | | | | | | | |
| March, 15 | BioDyn | --- | Biodynamic spraying | Horn manure (12g/5l) | 40 | --- | l/ha |
| March, 15 | Baseline sampling (T0) | | | | | | |
| March, 17 | BioDyn | --- | Slurry application | --- | 20 | --- | m^3^/ha |
| March,17 | --- | ConMin | N-Fertilisation | Ammonium sulphate nitrate | --- | 60 | kg N/ha |
| March, 20 | --- | ConMin | Herbicide application with growth regulators | HusarOD/ Mondera/ Cycocel extra | --- | 0.1/1/1.5 | l/ha |
| March, 21/22 | Rainout-shelter set-up | | | | | | |
| March, 28 | BioDyn | --- | Hoeing and flex time weeding | --- | --- | 1 | Passage |
| March, 31 | BioDyn | --- | Biodynamic spraying | Horn silica (1g/5l) | --- | 40 | l/ha |
| April, 11 | --- | ConMin | Fungicide application | Pronto plus | --- | 1.5 | l/ha |
| April, 12 | BioDyn | --- | Slurry application | Slurry tank with dosimate | 20 | --- | m^3^/ha |
| April, 12 | --- | ConMin | N-fertilisation | Calcium ammonium nitrate | --- | 40 | kg N/ha |
| April, 19 | BioDyn | --- | Biodynamic spraying | Horn silica (1g/5l) | --- | 40 | l/ha |
| April, 20 | First sampling date (T1) | | | | | | |
| May, 17 | Second sampling date (T2) | | | | | | |
| May, 17 | --- | ConMin | N-fertilisation | Calcium ammonium nitrate | --- | 40 | kg N/ha |
| May, 30 | --- | ConMin | Fungicide/Insecticide application | Aviator Xpro/Miros FL/Audienz | --- | 1/1/0.1 | l/ha |
| June, 20 | BioDyn | --- | Biodynamic spraying | Horn silica (1g/5l) | --- | 40 | l/ha |
| June, 20 | Third sampling date (T3) | | | | | | |

| **primer** | **target region** | **sequence [5’-3’]** | **PCR details** | **reference** |
| --- | --- | --- | --- | --- |
| 515F | V3-V5 hypervariable region of the bacterial 16S rRNA gene | GTGYCAGCMGCCGCGGTAA | Total volume: 20 µl  DNA template concentration: 1.0 ng/μl  Polymerase: Phusion Hot Start High  Fidelity Polymerase (Thermo Fisher Scientific, Waltham, USA)  Cycling conditions:  Initial denaturation at 98 °C (30 s) followed by 25 cycles of denaturation at 98 °C (10 s), annealing at 60 °C (30 s), extension at 72 °C (15 s) and a final extension step at 72 °C for 8 min | Parada et al., 2016 |
| 926R |  | CCGYCAATTYMTTTRAGTTT |  |  |

Table S2. Primer sequences and cycling conditions used to generate amplicons of prokaryotic soil communities by amplicon-based sequencing using the Illumina® MiSeq system.

reference: Parada, A. E., Needham, D. M., & Fuhrman, J. A. (2016). Every base matters: Assessing small subunit rRNA primers for marine microbiomes with mock communities, time series and global field samples. Environmental Microbiology, 18(5), 1403–1414. https://doi.org/10.1111/1462-2920.13023

Table S3. Primer details and cycling conditions used to generate amplicons to profile fungal communities by next-generation sequencing using the PacBio sequencing platform. ITS: internal transcribed spacer, BUP primer: Barcoded universal primer (Pacific Biosciences of California, Part number 100-466-100). Sequences in green indicate universal tag later used as target for the barcoding PCR (PCR2), sequences in blue indicate target specific sequences. [AmC6]: 5’ block

| **PCR** | **primer** | **target region** | **sequence [5’-3’]** | **reaction mixture** | **cycling program** | **reference** |
| --- | --- | --- | --- | --- | --- | --- |
| **1** | 1389F | Full ITS | **[AmC6]** **GCA GTC GAA CAT GTA GCT GAC TCA GGT CAC TTG TAC ACA CCG CCC** | Total volume: 20 µl  Reaction mixture: 5µl of HOT FIREPol Blend Master Mix (Solis Biodyne, Tartu, Estonia), 2.5 µM of each primer, 1 µl of DNA extract or water for the negative control, and sterile distilled water. | 15 min at 95°C; 20 x (30s at 95 s, 30 s at 55°C, 1 min at 72°C); 10 min at 72°C | Tedersoo et al., 2015, 2018  https://www.pacb.com/wp-content/uploads/2015/09/Procedure-and-Checklist-Preparing-SMRTbell-Libraries-PacB-Barcoded-Universal-Primers.pdf |
|  | ITS4ngsUni |  | **[AmC6] TGG ATC ACT TGT GCA AGC ATC ACA TCG TAG** **CCT SCS CTT ANT DAT ATG C** |  |  |  |
| **2** | BUP primers | Universal tag of PCR1 products | NA | Total volume: 20 µl containing 5 µl of HOT FIREPol Blend Master Mix (Solis Biodyne, Tartu, Estonia), 2 µl of BUP primer, 1 µl of purified PCR1 product/negative control and sterile distilled water | 15 min at 95°C; 20 x (30s at 95 s, 30 s at 64°C, 1 min at 72°C); 10 min at 72°C | https://www.pacb.com/wp-content/uploads/2015/09/Procedure-and-Checklist-Preparing-SMRTbell-Libraries-PacB-Barcoded-Universal-Primers.pdf |

References: Tedersoo, L., Anslan, S., Bahram, M., Põlme, S., Riit, T., Liiv, I., … Abarenkov, K. (2015). Shotgun metagenomes and multiple primer pair-barcode combinations of amplicons reveal biases in metabarcoding analyses of fungi. *MycoKeys*, *10*, 1–43. <https://doi.org/10.3897/mycokeys.10.4852>; Tedersoo, L., Tooming-Klunderud, A., & Anslan, S. (2018). PacBio metabarcoding of Fungi and other eukaryotes: errors, biases and perspectives. *New Phytologist*, *217*(3), 1370–1385. https://doi.org/10.1111/nph.14776

Table S4. Soil characteristics in the biodynamic (BioDyn) and the conventional-mineral (ConMin) farming system and differences between the farming systems assessed one week before setting up the experiment. Given are medians of the posterior distribution with 95% credible intervals (CrIs).

| parameter | BioDyn  mean (95% CrI) | ConMin  mean (95% CrI) | Difference between BioDyn and ConMin  mean (95% CrI) |
| --- | --- | --- | --- |
| pH (H_2_O) | 6.62 (6.12, 7.10) | 6.03 (5.52, 6.53) | +0.59 (0.14, 1.01) |
| C_tot_ [%] | 1.60 (1.36, 1.86) | 1.27 (1.03, 1.53) | +0.33 pp (0.16, 0.49) |
| N_tot_ [%] | 0.17 (0.14, 0.20) | 0.13 (0.10, 0.16) | +0.04 pp (0.02, 0.06) |
| WHC (0-10 cm) [%] | 42.0 (38.1, 45.9) | 38.6 (34.8, 42.2) | +3.4 pp (0.0, 6.9) |
| WHC (10-20 cm) [%] | 40.1 (36.3, 44.0) | 36.9 (33.1, 40.8) | +3.2 pp (-0.1, 6.7) |
| bulk density (0-10 cm) [g/cm^3^] | 1.18 (1.08, 1.29) | 1.22 (1.11, 1.32) | -0.04 (-0.12, 0.05) |
| bulk density (10-20 cm) [g/cm^3^] | 1.21 (1.11, 1.32) | 1.25 (1.14, 1.35) | -0.04 (-0.12, 0.05) |
| PO_4_-P [µg/g DW] | 1.08 (0.86, 1.30) | 1.03 (0.83, 1.26) | +0.04 (-0.15, 0.24) |
| P [mg/g DW] | 0.80 (0.66, 0.94) | 0.82 (0.68, 0.97) | -0.03 (-0.11, 0.06) |

*pH (H_2_O): soil pH measured in water, C_tot_: total soil carbon (in the field C_tot_ is all organic carbon); N_tot_: total soil nitrogen; WHC: water holding capacity in undisturbed soil samples; P: phosphorous, PO_4_-P: Orthophosphate as phosphorus*

Table S5. Details on total number of sequences (median and range) and unique operational taxonomic units (OTUs) for the bacterial and fungal data set before and after filtering rare OTUs (fewer than 20 reads occurring in fewer than 5% of the samples).

|  |  | sequences | | unique OTUs | |
| --- | --- | --- | --- | --- | --- |
|  |  | bacteria | fungi | bacteria | fungi |
| before filtering | total | 799,203 | 113,224 | 3,154 | 454 |
|  | median | 10,274 | 1,544 |  |  |
|  | range | 6,348-26,884 | 454-2,915 |  |  |
| after filtering | total | 793,737 | 109,020 | 2,763 | 357 |
|  | median | 10,202 | 1,491 |  |  |
|  | range | 6,287-26,700 | 450-2,837 |  |  |
| kept [%] | | 99 | 96 | 88 | 79 |

Table S6. Pairwise Adonis investigating the differences in community composition between the three sampling dates (time) for (A) bacteria and (B) fungi. Data derive from amplicon-based sequencing of bacterial (16SrRNA) and fungal (ITS) marker genes. Factor time: 4 (T1), 8 (T2) and 13 (T3) weeks after rainout-shelter set-up.

| **(A)** bacteria | | | | | | |  |
| --- | --- | --- | --- | --- | --- | --- | --- |
| comparison | source of variation | degrees of freedom | sum of squares | mean squares | pseudo  F-value | R^2^ | Pr(>F) |
| T1 vs T2 | time | 1 | 0.105 | 0.105 | 1.652 | 0.035 | 0.094 |
|  | residuals | 46 | 2.922 | 0.064 | --- | 0.965 | --- |
|  | total | 47 | 3.027 | --- | --- | 1.000 | --- |
| T1 vs T3 | time | 1 | 0.237 | 0.237 | 4.042 | 0.081 | 0.002 |
|  | residuals | 46 | 2.701 | 0.059 | --- | 0.919 | --- |
|  | total | 47 | 2.938 | --- | --- | 1.000 | --- |
| T2 vs T3 | time | 1 | 0.322 | 0.322 | 5.063 | 0.099 | 0.001 |
|  | residuals | 46 | 2.921 | 0.064 | --- | 0.901 | --- |
|  | total | 47 | 3.243 | --- | --- | 1.000 | --- |
|  |  | | | | | |  |
| **(B)** fungi | | | | | | |  |
| comparison | source of variation | degrees of freedom | sum of squares | mean squares | pseudo  F-value | R^2^ | Pr(>F) |
| T1 vs T2 | time | 1 | 0.089 | 0.089 | 0.624 | 0.013 | 0.965 |
|  | residuals | 46 | 6.559 | 0.143 | --- | 0.987 | --- |
|  | total | 47 | 6.648 | --- | --- | 1.000 | --- |
| T1 vs T3 | time | 1 | 0.334 | 0.334 | 2.407 | 0.050 | 0.003 |
|  | residuals | 46 | 6.387 | 0.139 | --- | 0.950 | --- |
|  | total | 47 | 6.722 | --- | --- | 1.000 | --- |
| T2 vs T3 | time | 1 | 0.370 | 0.370 | 2.697 | 0.055 | 0.001 |
|  | residuals | 46 | 6.318 | 0.137 | --- | 0.945 | --- |
|  | total | 47 | 6.688 | --- | --- | 1.000 | --- |

Table S7. Overview of the environmental variables subjected to the *envfit* function. Given is the correlation strength (r^2^) between variables and bacterial and fungal ordination scores along with the corresponding p-values. Variables with r^2^ values > 0.4 (in bold) were plotted onto the constraint ordinations (Figure 6). All variables were z-transformed before analysis and permutations restricted within field blocks.

| environmental variables | (A) bacteria | | (B) fungi | |
| --- | --- | --- | --- | --- |
|  | r^2^ | p | r^2^ | p |
| volumetric soil water content [%] | **0.639** | 0.001 | **0.575** | 0.001 |
| soil pH (H_2_O) | **0.695** | 0.001 | **0.695** | 0.001 |
| soil carbon to nitrogen ratio | 0.292 | 0.001 | 0.292 | 0.001 |
| total soil nitrogen content [%] | **0.695** | 0.001 | **0.695** | 0.001 |
| total soil carbon content [%] | **0.737** | 0.001 | **0.737** | 0.001 |
| total sand content [%] | 0.001 | 0.965 | 0.001 | 0.964 |
| total silt content [%] | 0.000 | 0.995 | 0.000 | 0.995 |
| total clay content [%] | 0.005 | 0.781 | 0.005 | 0.796 |
| weed cover [%] | **0.527** | 0.001 | **0.546** | 0.001 |
| root dry weight [g/m^2^] | 0.128 | 0.008 | 0.100 | 0.026 |
| soil water holding capacity [%] | **0.406** | 0.001 | **0.405** | 0.001 |
| soil bulk density [g/cm^3^] | 0.139 | 0.001 | 0.139 | 0.001 |

Table S8. Bacterial indicator species identified in the biodynamic farming system. In total, 793,737 sequences were subjected to the indicator species analysis. Only taxa with a point biserial correlation coefficient (corr. coef) >0.6 and a p-value <0.01 are shown. Number of sequences (No of sequences) are the total sequence counts of the indicator OTUs in the biodynamic farming system.


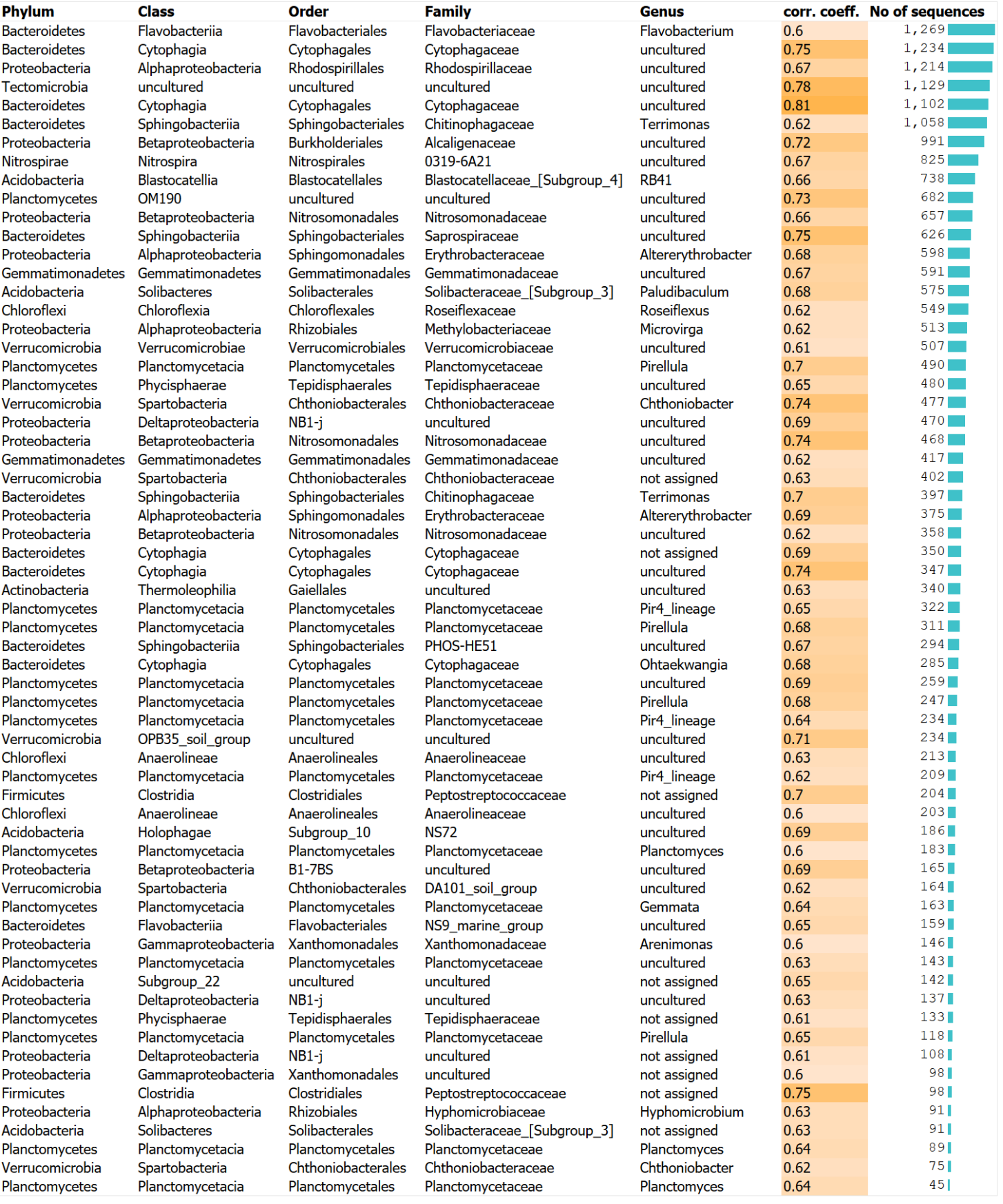


Table S9. Bacterial indicator species identified in the conventional farming system. In total, 793,737 sequences were subjected to the indicator species analysis. Only taxa with a point biserial correlation coefficient (corr. coef) >0.6 and a p-value <0.01 are shown. Number of sequences (No of sequences) are the total sequence counts of the indicator OTUs in the conventional farming system.


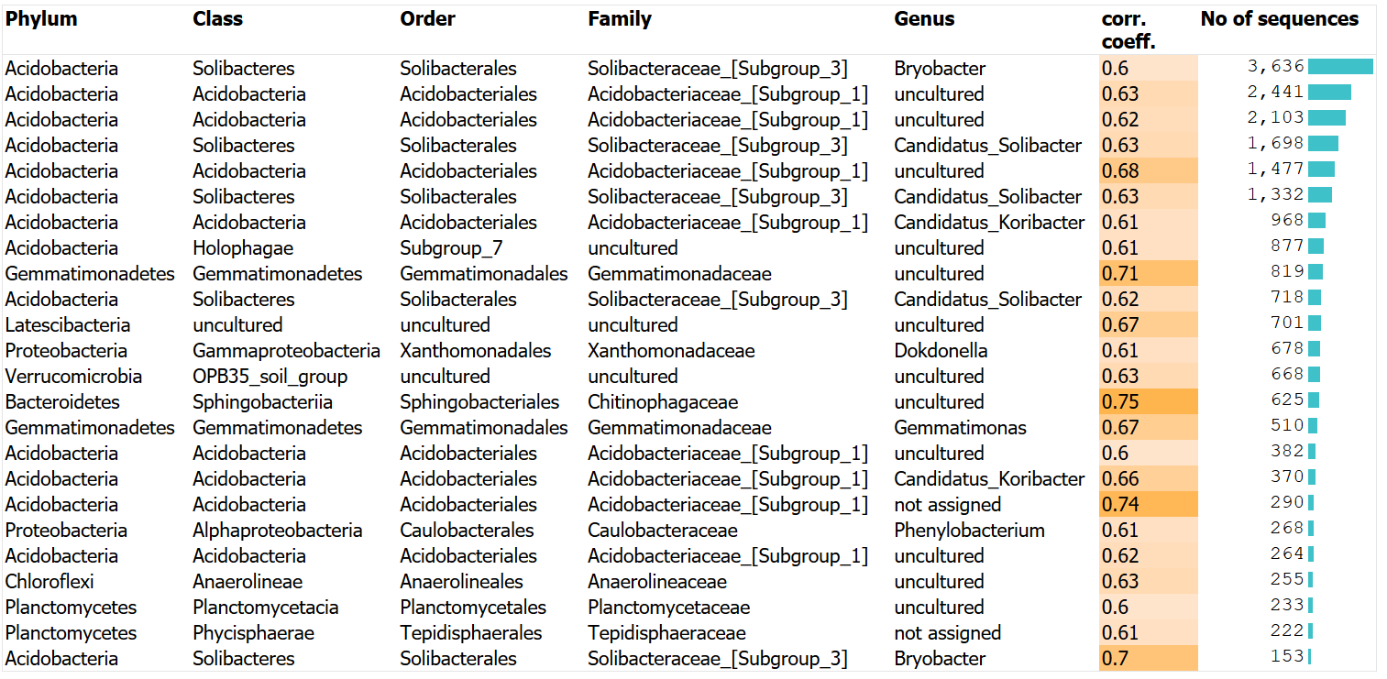


Table S10. Fungal indicator species identified in the BioDyn and the ConMin farming system. In total, 109,020 sequences were subjected to the indicator species analysis. Only taxa with a point biserial correlation coefficient (corr. coef) >0.6 and a p-value <0.01 are shown. Number of sequences (No of sequences) are the total sequence counts of the indicator OTUs in the given farming system.


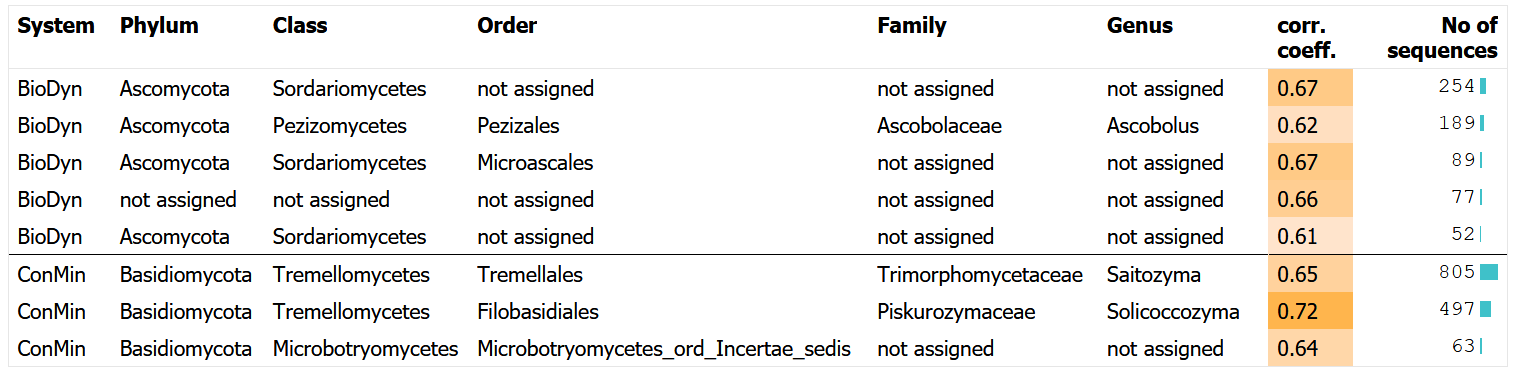


Table S11. Linear regression model assessing the effect of AM fungi abundance on plant related parameters (straw and grain yield, total shoot biomass, plant height) at the last sampling date (T3). For each farming system, a separate model was applied. Coefficients for the regression slope are provided as median of the posterior distribution together with the slope’s upper (u-95% CrI) and lower 95% (l-95% CrI) credible boundaries. Factor system: biodynamic farming system (BioDyn), conventional farming system with pure mineral fertilisation (ConMin)

| farming system | plant parameter | regression slope (ß) | | |
| --- | --- | --- | --- | --- |
|  |  | median | l-95% CrI | u-95% CrI |
| ConMin | straw yield [t/ha] | -0.17 | -0.48 | 0.14 |
|  | grain yield [t/ha] | -0.14 | -0.36 | 0.08 |
|  | total shoot [t/ha] | -0.31 | -0.84 | 0.21 |
|  | plant height [cm] | -0.50 | -1.41 | 0.40 |
| BioDyn | straw yield [t/ha] | -0.02 | -0.20 | 0.15 |
|  | grain yield [t/ha] | -0.02 | -0.13 | 0.07 |
|  | total shoot [t/ha] | -0.05 | -0.32 | 0.20 |
|  | plant height [cm] | 0.05 | -0.33 | 0.44 |

## Supplementary Figures


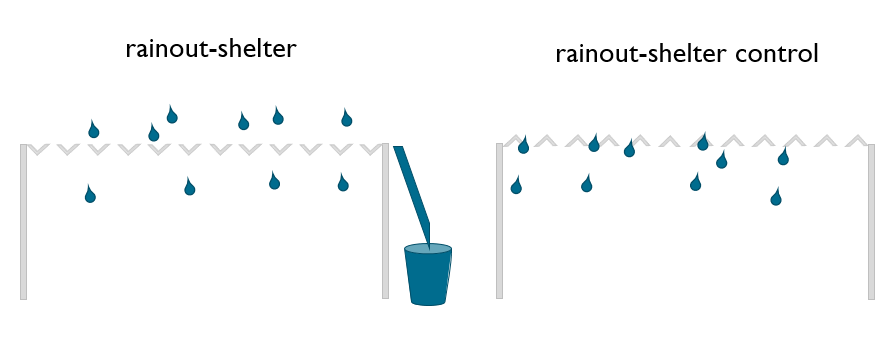


Figure S1. Schematic drawings of the partial rainout-shelter (left) and the rainout-shelter control (right). For both shelter types, V-shaped acrylic glass panels were mounted on metal frames; for the rainout-shelter control, the panels were turned over (no active interception of precipitation), the rainout-shelter is supplied with a rain gutter, so that the intercepted precipitation is collected in a rain barrel. More details on the shelters are available in Kundel et. al 2018 (Front. Environ. Sci., 22 March 2018 | <https://doi.org/10.3389/fenvs.2018.00014>).


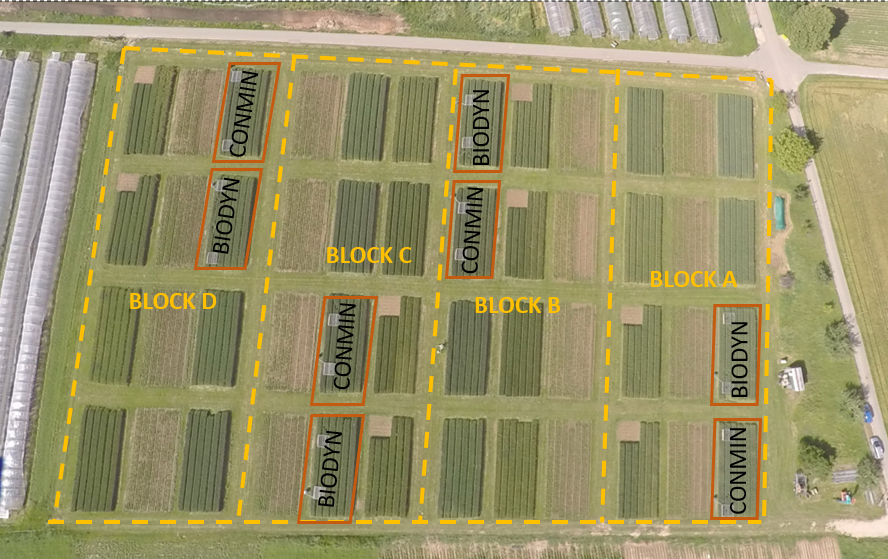


Figure S2. The “DOK” long-term comparison trial for different farming systems was used as platform for the current experiment. Each farming system (BioDyn, ConMin) is represented by four field plots (brown outline). The field plots are nested within field blocks (dashed, yellow outline). The three levels of the drought treatment (Control: no rainout-shelter, rainout-shelter control and rainout-shelter; **not included in this overview**) are referred to as subplots and nested within the field plots. BioDyn: biodynamic farming system; ConMin: conventional farming system with pure mineral fertilisation.


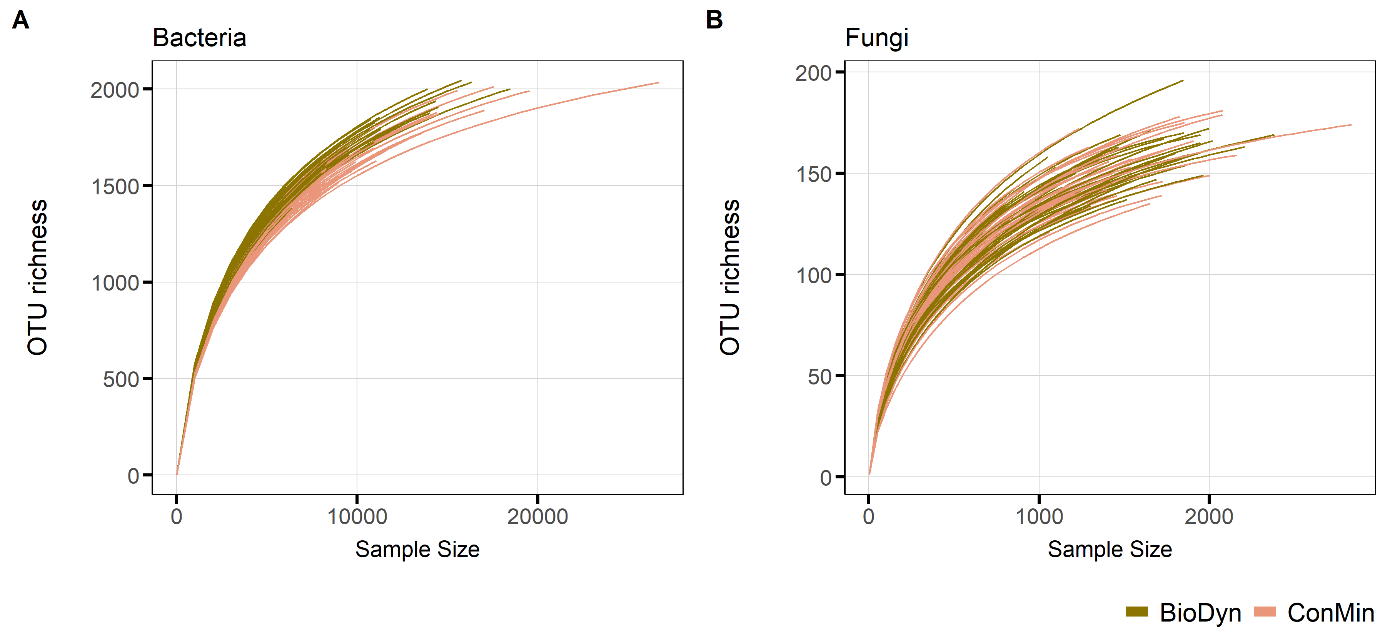


Figure S3. Rarefaction curves for (A) bacterial and (B) fungal sequencing data after removing non bacterial and non-fungal sequences and filtering low abundant (fewer than 20 reads occurring in fewer than 5% of the samples) operational taxonomic units (OTUs).


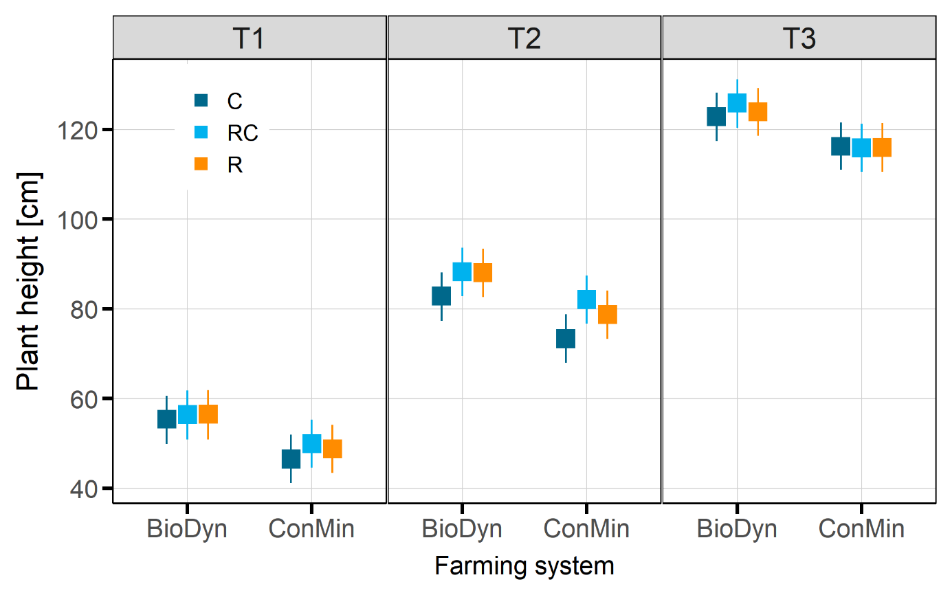


Figure S4. Plant height (cm). Data are medians of the posterior distribution with 95% credible intervals (CrIs). Factor system: biodynamic farming system (BioDyn), conventional farming system with pure mineral fertilisation (ConMin); factor drought*:* control (C, no shelter), rainout-shelter control (RC), rainout-shelter (R); factor time: 4 (T1), 8 (T2) and 13 (T3) weeks after rainout-shelter set-up.


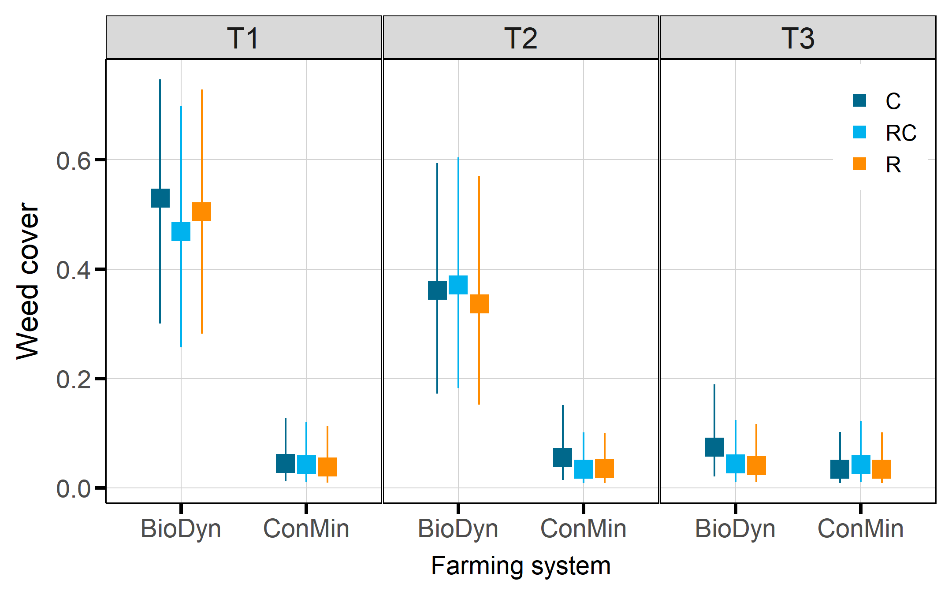


Figure S5. Proportion of soil surface covered with weeds. Data are medians of the posterior distribution with 95% credible intervals (CrIs). Factor system: biodynamic farming system (BioDyn), conventional farming system with pure mineral fertilisation (ConMin); factor drought*:* control (C, no shelter), rainout-shelter control (RC), rainout-shelter (R); factor time: 4 (T1), 8 (T2) and 13 (T3) weeks after rainout-shelter set-up.
