# Supplementary material for: Effects of simulated drought on biological soil quality, microbial diversity and yields under long-term conventional and organic agriculture
Source: FEMS Microbiol Ecol. 2020 Oct 5;96(12):fiaa205. doi: 10.1093/femsec/fiaa205 (PMC7705324; doi:10.1093/femsec/fiaa205)
Supplement: fiaa205_Supplemental_Files [file fiaa205_supplemental_files.zip › Supporting_file_01.html]

p327 Run190923


# p327 Run190923

#### Jean-Claude Walser

#### 11/26/2019

# 1 Workflow: p327\_run190417\_ITS\_PacBio\_CSS

## 1.1 Run-Info

```
## =======================================================================================
## | A0 | Run Info
## =======================================================================================

## Project: p327
## Run: run190417 (Pacbio_soilclim)
## Reads: ITS AmpSEq with PacBio (ccs.fastq)
## Provider:
## N(samples)= 96
```

## 1.2 Workflow Summary

The data preparation workflow was run on the HPC EULER, ETH Zurich. The workflow prep steps to optimise resource management:

- [A] Quality control
- [B] GZ-to-FQ
- [C] Primer Trimming (in-silico PCR with size selection)
- [D] Quality filtering and Size Selection
- [E] OTU clustering / amplicon sequence variants (ASV)
- [F] Annotation predictions

## 1.3 A - Quality Control & Parameter Evaluation

```
# ==================================================
# Parameter Report
# Sample: p327/Pacbio_soilclim/ccs.f*q
# --------------------------------------------------
# Primer
# 1389F: TTGTACACACCGCCC
# ITS4ngsUni: CCTSCSCTTANTDATATGC
# --------------------------------------------------
# In-silico PCR with increasing error rate
#
# Number of total reads: 283,315
# Mismatche 0 = 122,563 (+) / 127,011 (-) Amplicons
# Mismatche 1 = 128,137 (+) / 132,662 (-) Amplicons
# Mismatche 2 = 130,019 (+) / 134,492 (-) Amplicons
# Mismatche 3 = 131,486 (+) / 135,931 (-) Amplicons
# Mismatche 4 = 135,324 (+) / 139,768 (-) Amplicons
# Mismatche 5 = 158,698 (+) / 163,467 (-) Amplicons
#
# --------------------------------------------------
# Fragment Size Distribution
#
# Maximum value 1901.000000
# 480  286
# 520 ** 2356
# 560 *** 3646
# 600 * 1743
# 640 ************ 17331
# 680 ************************************************************ 84480
# 720 ******************** 28791
# 760 **************************** 38889
# 800 ************************************** 52821
# 840 ********** 13477
# 880 ****** 8828
# 920 *** 3992
# 960 ** 3291
# <minVal or >=1000 *** 4580
# ==================================================
```

## 1.4 B - GZ-to-QC (on scratch)

```
==================================================
Project: p327
Run: run190417
Group: soilclim
N(sample): 96
--------------------------------------------------
START_GZ-2-FQ: 16:43:50 18/04/2019
END_GZ-2-FQ: 16:43:55 18/04/2019
==================================================
```

## 1.5 C - In-Silico PCR

```
==================================================
Project: p327
Run: run190417
Group: soilclim
--------------------------------------------------
usearch v11.0.667_i86linux64
Amplicon range: 300-1200
Number of mis-matches: 2
Coverage: full-length
Wildcards enabled: IUPAC codes
--------------------------------------------------
# Primer
# 1389F: TTGTACACACCGCCC
# ITS4ngsUni: CCTSCSCTTANTDATATGC
--------------------------------------------------
START_In-Silico_PCR: 14:29:56 02/05/2019
END_In-Silico_PCR: 14:37:43 02/05/2019
==================================================
```

## 1.6 D - Size Selection and Quality Filtering

```
==================================================
Project: p327
Run: run190417
Group: soilclim
--------------------------------------------------
PRINSEQ-lite 0.20.4
Size Range: 450-1200
GC Range: 30-70
Min Q Mean: 20
Number of Ns: 0
Low Complexity: dust / 30
--------------------------------------------------
START_QF: 14:37:56 02/05/2019
END_QF: 14:41:03 02/05/2019
--------------------------------------------------
Statistic Report
START_Reporting: 14:41:03 02/05/2019
END_Reporting: 14:41:30 02/05/2019
--------------------------------------------------
Encode Reads
START_Encoding: 14:41:30 02/05/2019
Number of reads: 272239 / 272239
END_Encoding: 14:41:36 02/05/2019
==================================================
```

Read preparation report:

| Sample | Raw | Primer | Clean | MeanLength |
| --- | --- | --- | --- | --- |
| S0001 | 2783 | 2503 | 2503 | 705.871 |
| S0002 | 2487 | 2388 | 2366 | 734.24 |
| S0003 | 2234 | 2149 | 2146 | 738.255 |
| S0004 | 1789 | 1788 | 1788 | 752.389 |
| S0005 | 2674 | 2573 | 2567 | 718.818 |
| S0006 | 4229 | 4095 | 4070 | 725.606 |
| S0007 | 3176 | 2976 | 2969 | 720.795 |
| S0008 | 3146 | 3052 | 3036 | 722.699 |
| S0009 | 3693 | 3796 | 3786 | 746.425 |
| S0010 | 2462 | 2292 | 2284 | 761.36 |
| S0011 | 1948 | 1892 | 1873 | 718.284 |
| S0012 | 2497 | 2427 | 2414 | 724.632 |
| S0013 | 2737 | 2530 | 2521 | 697.408 |
| S0014 | 2345 | 2274 | 2265 | 717.782 |
| S0015 | 3485 | 3266 | 3244 | 747.998 |
| S0016 | 3123 | 2997 | 2972 | 721.626 |
| S0017 | 1873 | 1830 | 1822 | 715.681 |
| S0018 | 2481 | 2415 | 2403 | 714.903 |
| S0019 | 3095 | 3093 | 3087 | 747.967 |
| S0020 | 2333 | 2291 | 2287 | 746.543 |
| S0021 | 3096 | 2749 | 2746 | 720.104 |
| S0022 | 3171 | 3096 | 3084 | 717.799 |
| S0023 | 3587 | 3405 | 3366 | 722.823 |
| S0024 | 2657 | 2459 | 2449 | 739.256 |
| S0025 | 1939 | 1872 | 1870 | 735.911 |
| S0026 | 2238 | 2068 | 2066 | 698.121 |
| S0027 | 3724 | 3367 | 3366 | 700.455 |
| S0028 | 3726 | 3537 | 3533 | 726.218 |
| S0029 | 4391 | 4049 | 4038 | 742.99 |
| S0030 | 3334 | 3276 | 3262 | 725.495 |
| S0031 | 1708 | 1705 | 1699 | 722.178 |
| S0032 | 3963 | 3800 | 3790 | 720.173 |
| S0033 | 3543 | 3495 | 3486 | 721.832 |
| S0034 | 2942 | 2718 | 2711 | 747.642 |
| S0035 | 2478 | 2400 | 2357 | 725.115 |
| S0036 | 2606 | 2448 | 2433 | 740.136 |
| S0037 | 2758 | 2670 | 2665 | 713.42 |
| S0038 | 1811 | 1727 | 1721 | 740.959 |
| S0039 | 3131 | 3131 | 3129 | 731.713 |
| S0040 | 3106 | 3141 | 3133 | 720.221 |
| S0041 | 3082 | 3045 | 3042 | 713.076 |
| S0042 | 2607 | 2369 | 2368 | 771.626 |
| S0043 | 1820 | 1839 | 1837 | 733.94 |
| S0044 | 2762 | 2663 | 2661 | 747.354 |
| S0045 | 3541 | 3463 | 3460 | 747.262 |
| S0046 | 2692 | 2659 | 2655 | 734.191 |
| S0047 | 4271 | 4108 | 4103 | 718.727 |
| S0048 | 2651 | 2654 | 2643 | 720.037 |
| S0049 | 3726 | 3682 | 3676 | 728.196 |
| S0050 | 2407 | 2286 | 2281 | 742.752 |
| S0051 | 3605 | 3443 | 3438 | 715.552 |
| S0052 | 2976 | 2807 | 2799 | 714.251 |
| S0053 | 3259 | 3048 | 3040 | 715.538 |
| S0054 | 2444 | 2341 | 2337 | 736.107 |
| S0055 | 2495 | 2433 | 2421 | 732.467 |
| S0056 | 1598 | 1598 | 1593 | 716.974 |
| S0057 | 2726 | 2733 | 2727 | 718.776 |
| S0058 | 3964 | 3575 | 3567 | 760.698 |
| S0059 | 2873 | 2706 | 2696 | 750.898 |
| S0060 | 2844 | 2664 | 2658 | 714.419 |
| S0061 | 2375 | 2222 | 2219 | 719.644 |
| S0062 | 3457 | 3166 | 3158 | 725.89 |
| S0063 | 3316 | 3101 | 3095 | 700.788 |
| S0064 | 3324 | 3157 | 3151 | 710.7 |
| S0065 | 2020 | 1955 | 1951 | 719.978 |
| S0066 | 2856 | 2758 | 2755 | 707.634 |
| S0067 | 2731 | 2684 | 2675 | 744.351 |
| S0068 | 3067 | 3002 | 2994 | 735.033 |
| S0069 | 2408 | 2406 | 2402 | 731.69 |
| S0070 | 1023 | 1001 | 998 | 751.099 |
| S0071 | 3303 | 3167 | 3143 | 726.652 |
| S0072 | 3831 | 3801 | 3795 | 735.261 |
| S0073 | 2743 | 2699 | 2674 | 722.806 |
| S0074 | 2928 | 2797 | 2796 | 751.739 |
| S0075 | 2778 | 2672 | 2662 | 710.211 |
| S0076 | 4499 | 4291 | 4271 | 730.949 |
| S0077 | 3290 | 3122 | 3115 | 725.389 |
| S0078 | 2998 | 2952 | 2943 | 727.272 |
| S0079 | 2920 | 2680 | 2650 | 722.087 |
| S0080 | 4327 | 4144 | 4133 | 718.675 |
| S0081 | 3255 | 3181 | 3173 | 716.64 |
| S0082 | 4003 | 3791 | 3781 | 765.974 |
| S0083 | 3175 | 2952 | 2945 | 754.809 |
| S0084 | 2067 | 2591 | 2583 | 739.615 |
| S0085 | 2069 | 1985 | 1972 | 728.863 |
| S0086 | 2436 | 2320 | 2315 | 719.359 |
| S0087 | 3344 | 3119 | 3112 | 713.535 |
| S0088 | 3246 | 3193 | 3188 | 721.388 |
| S0089 | 3152 | 3049 | 3043 | 731.035 |
| S0090 | 3508 | 3437 | 3431 | 717.218 |
| S0091 | 3158 | 3099 | 3081 | 735.931 |
| S0092 | 3408 | 3054 | 3045 | 760.404 |
| S0093 | 3667 | 3745 | 3733 | 767.358 |
| S0094 | 4384 | 4539 | 4529 | 740.593 |
| S0095 | 2883 | 2836 | 2826 | 752.629 |
| S0096 | 2531 | 2600 | 2593 | 733.611 |

## 1.7 E - UPARSE & UNOISE

```
================================================================================
Project: p327
Run: run190417
Group: soilclim
File: p327_run190417_soilclim_bc.fasta
--------------------------------------------------------------------------------
UPARSE & UNOISE3
usearch v11.0.667_i86linux64
--------------------------------------------------------------------------------
START: 14:41:57 02/05/2019
  ▶ Deduplicate Amplicons
  --------------------------------------------------------------------------------
  Dereplicates amplicons to obtain unique amplicons.
  Determin error rates of amplicon reads. 
  --------------------------------------------------------------------------------
  ▶ Cluster OTU (97%)
  --------------------------------------------------------------------------------
  Clusters OTU at 97% using the UPARSE-OTU algorithm.
  Number of OTUs: 2277
  --------------------------------------------------------------------------------
  ▶ Unoise3
  --------------------------------------------------------------------------------
  Uses the UNOISE algorithm to perform denoising (error-correction) of amplicon reads.
  Number of ZOTUs: 1164
  --------------------------------------------------------------------------------
  ▶ Additional Clustering
  --------------------------------------------------------------------------------
  Clusters ZOTUs at different identity levels (i.e. 97%,98% and 99%).
  Number of ZOTUs 99%: 851
  Number of ZOTUs 98%: 781
  Number of ZOTUs 97%: 730
  --------------------------------------------------------------------------------
  ▶ Count Table
  --------------------------------------------------------------------------------
  Generates OTU count tables by mapping reads to OTUs.
  --------------------------------------------------------------------------------
  ▶ ZOTU Table Report
  --------------------------------------------------------------------------------
  Creates a report/summary from an OTU table.
  --------------------------------------------------------------------------------
  ▶ Distance matrices
  --------------------------------------------------------------------------------
  Pairwise distances between (Z)OTU sequences.
  --------------------------------------------------------------------------------
  ▶ Octave plots
  --------------------------------------------------------------------------------
  Octave plots with low-abundance (Z)OTUs and cross-talk information.
  --------------------------------------------------------------------------------
  ▶ Alignment and Tree
  --------------------------------------------------------------------------------
  Creates multiple sequences alignment and clustered tre files.
  The trees will be very approximate in both cases.
  --------------------------------------------------------------------------------
  ▶ Alpha Diversity - Evenness Berger-Parker
  --------------------------------------------------------------------------------
  Evenness based on Berger-Parker.
  A value close to 1 indicates that a single large OTU dominates the sample,
  small values indicate that the reads are distributed over many OTUs.
  --------------------------------------------------------------------------------
  ▶ Rarefaction
  --------------------------------------------------------------------------------
  Calculates a rarefaction curve for an alpha diversity metric.
  The output file can easily be loaded into a spreadsheet or R for generating figures.
  --------------------------------------------------------------------------------
  ▶ Beta Diversity
  --------------------------------------------------------------------------------
  Calculates beta diversity metrics from an OTU table.
  --------------------------------------------------------------------------------
  ▶ Uncross
  --------------------------------------------------------------------------------
  Detects and filters cross-talk (sample mis-assignment) in a OTU table using the UNCROSS algorithm.
  --------------------------------------------------------------------------------
END: 15:48:46 02/05/2019
================================================================================
```

## 1.8 F - Taxonomic Assignment Predictions

```
==========================================================================================
Project: p327
Run: run190417
Group: soilclim
==========================================================================================
SINTAXv11.0.667_i86linux64
Database: ITS/UNITE_UTAX_V7.2_10.10.2017.fasta
Tax filter: 0.85
Workflow Summary:
  (a) Adjust DB according to amplicons (usearch_global; strand both; id 0.7)
  (b) Assign taxa (sintax; strand both; sintax_cutoff 0.7)
  (c) Reformat tax information for phyloseq import
  (d) Combine count table and taxa
START_Sintax: 15:48:58 02/05/2019
  Start_F1_trimDB: 15:48:58 02/05/2019
  End_F1_trimDB: 16:20:16 02/05/2019
  Start_F1_Unique_records: 16:20:16 02/05/2019
  End_F1_Unique_records: 16:20:19 02/05/2019
  Start_F1_Build_UPD: 16:20:20 02/05/2019
  End_F1_Build_UPD: 16:20:28 02/05/2019
  Start_F2_for_OTU: 16:20:28 02/05/2019
  End_F2_for_OTU: 16:20:55 02/05/2019
  Start_F2_for_ZOTU: 16:20:55 02/05/2019
  End_F2_for_ZOTU: 16:21:31 02/05/2019
  Start_TaxSummary: 16:21:31 02/05/2019
  End_TaxSummary: 16:21:31 02/05/2019
  Start_ChimeraCheck: 16:21:31 02/05/2019
  End_ChimeraCheck: 16:24:11 02/05/2019
END_Sintax: 16:24:11 02/05/2019
==========================================================================================
```

## 1.9 G - Summary

```
## (Z)OTU Summary:

# Number of OTUs:       2,277
# Number of ZOTUs:      1,164
# Number of ZOTUs 99%:    851
# Number of ZOTUs 98%:    781
# Number of ZOTUs 97%:    730

## Annotation Level Summary:

# OTU:
# d: ********************** 212
# p: ************************************************************ 582
# c: ************************************************ 466
# o: *********** 110
# f: ************ 113
# g: ***************************************** 397
# s: ***************************************** 397

# ZOTU:
# d: ************************************************************ 545
# p: ******* 62
# c: *********** 97
# o: **************** 144
# f: ************* 117
# g: **************** 143
# s: ****** 56

# ZOTU_c99:
# d: ************************************************************ 397
# p: ******* 49
# c: ********** 69
# o: *************** 101
# f: ************ 80
# g: ***************** 110
# s: ******* 45

# ZOTU_c98:
# d: ************************************************************ 371
# p: ****** 40
# c: *********** 70
# o: *************** 91
# f: *********** 67
# g: **************** 100
# s: ******* 42

# ZOTU_c97:
# d: ************************************************************ 344
# p: ******* 40
# c: ************ 66
# o: *************** 88
# f: ********** 60
# g: ***************** 96
# s: ****** 36
```

# 2 Workflow: p476\_run190923\_16S

## 2.1 Run-Info

```
## =======================================================================================
## | A0 | Run Info
## =======================================================================================

# Project: p476
# Run: run190923 
# Reads: MiSeq 16S PE250
# Provider: Jaak Truu

# N(samples) = 96
# N(reads) = 2,810,584
# N(MiSeqRuns) = 1
# Data Type = PE-244
# N(PhiX) = < 0.01%
# Merging rate = >95%
# Primer site = >90%
```

## 2.2 Workflow Summary

The data preparation workflow was run on the HPC EULER, ETH Zurich. The workflow prep steps to optimise resource management:

- [A] Quality control and parameter evaluation
- [B] Read end trimming and read merging
- [C] Primer trimming
- [D] Quality filtering
- [E] OTU clustering / amplicon sequence variants (ASV)
- [F] Annotation predictions

## 2.3 A - Quality Control & Parameter Evaluation

```
## Version: usearch v11.0.667_i86linux64

## Number of reads:
# N(R1)= 6'013'281
# N(R2)= 6'013'281

## =======================================================================================
## | A1 | PhiX Contamination (Subset)
## =======================================================================================

# sub1.fq:         1,000,000
# sub1_noPhiX.fq:  1,000,000 (d=0)
# sub2_noPhiX.fq:    999,994 (d=6)

## =======================================================================================
## | A2 | Read Length Distribution
## =======================================================================================

 30  7
 40  7
 50  12
 60  14
 70  6
 80  33
 90  18
100  20
110  21
120  32
130  91
140  187
150  241
160  257
170  499
180  320
190  368
200  497
210  429
220  420
230  393
240 ************************************************************ 2806712


## R1 Error Distribution
#
# 2810584 reads, max len 244, avg 243.9
# 
# Length         MaxEE 0.50         MaxEE 1.00         MaxEE 2.00
# ------   ----------------   ----------------   ----------------
#     50    2791760( 99.3%)    2807866( 99.9%)    2810535(100.0%)
#     60    2783327( 99.0%)    2805482( 99.8%)    2810184(100.0%)
#     70    2770985( 98.6%)    2802087( 99.7%)    2809215(100.0%)
#     80    2747631( 97.8%)    2795889( 99.5%)    2808152( 99.9%)
#     90    2729750( 97.1%)    2787924( 99.2%)    2806798( 99.9%)
#    100    2713287( 96.5%)    2779811( 98.9%)    2805297( 99.8%)
#    110    2696135( 95.9%)    2770252( 98.6%)    2803067( 99.7%)
#    120    2673254( 95.1%)    2755520( 98.0%)    2799493( 99.6%)
#    130    2652039( 94.4%)    2741086( 97.5%)    2794450( 99.4%)
#    140    2630855( 93.6%)    2725730( 97.0%)    2787808( 99.2%)
#    150    2605941( 92.7%)    2708147( 96.4%)    2779031( 98.9%)
#    160    2581120( 91.8%)    2689673( 95.7%)    2768564( 98.5%)
#    170    2557355( 91.0%)    2672601( 95.1%)    2758988( 98.2%)
#    180    2537361( 90.3%)    2657951( 94.6%)    2750220( 97.9%)
#    190    2514366( 89.5%)    2641134( 94.0%)    2740012( 97.5%)
#    200    2488246( 88.5%)    2621671( 93.3%)    2727332( 97.0%)
#    210    2460327( 87.5%)    2600959( 92.5%)    2714057( 96.6%)
#    220    2427870( 86.4%)    2577202( 91.7%)    2698746( 96.0%)
#    230    2361863( 84.0%)    2533732( 90.1%)    2672957( 95.1%)
#    240    2278295( 81.1%)    2478195( 88.2%)    2640161( 93.9%)

## R2 Error Distribution
#
# 2810584 reads, max len 244, avg 243.9
# 
# Length         MaxEE 0.50         MaxEE 1.00         MaxEE 2.00
# ------   ----------------   ----------------   ----------------
#     50    2713715( 96.6%)    2773477( 98.7%)    2807127( 99.9%)
#     60    2683481( 95.5%)    2753099( 98.0%)    2799177( 99.6%)
#     70    2652992( 94.4%)    2734815( 97.3%)    2789714( 99.3%)
#     80    2620241( 93.2%)    2714154( 96.6%)    2778557( 98.9%)
#     90    2572542( 91.5%)    2686498( 95.6%)    2764951( 98.4%)
#    100    2515739( 89.5%)    2650100( 94.3%)    2749977( 97.8%)
#    110    2458767( 87.5%)    2609590( 92.8%)    2731301( 97.2%)
#    120    2406319( 85.6%)    2568691( 91.4%)    2707629( 96.3%)
#    130    2358868( 83.9%)    2530450( 90.0%)    2682039( 95.4%)
#    140    2307120( 82.1%)    2488203( 88.5%)    2651778( 94.3%)
#    150    2247695( 80.0%)    2443579( 86.9%)    2620383( 93.2%)
#    160    2193076( 78.0%)    2401983( 85.5%)    2590834( 92.2%)
#    170    2109080( 75.0%)    2340155( 83.3%)    2548831( 90.7%)
#    180    1982888( 70.6%)    2251234( 80.1%)    2490386( 88.6%)
#    190    1829574( 65.1%)    2142482( 76.2%)    2419392( 86.1%)
#    200    1678841( 59.7%)    2031306( 72.3%)    2346074( 83.5%)
#    210    1484011( 52.8%)    1879183( 66.9%)    2243489( 79.8%)
#    220    1233127( 43.9%)    1679753( 59.8%)    2105794( 74.9%)
#    230     970715( 34.5%)    1451041( 51.6%)    1939935( 69.0%)
#    240     690346( 24.6%)    1170109( 41.6%)    1720979( 61.2%)

## =======================================================================================
## | A3 | Nucleotide Composition
## =======================================================================================

## R1
#
# File size 1.5G, 2.8M seqs, 685.6M letters and quals
# Lengths min 36, lo_quartile 244, median 244, hi_quartile 244, max 244
# Letter freqs G 35.0%, A 23.5%, C 20.8%, T 20.6%
# 0% masked (lower-case)
# ASCII_BASE=33
# EE mean 0.5; min 0.0, lo_quartile 0.0, median 0.1, hi_quartile 0.3, max 18.1

## R2
#
# File size 1.5G, 2.8M seqs, 685.6M letters and quals
# Lengths min 36, lo_quartile 244, median 244, hi_quartile 244, max 244
# Letter freqs C 31.6%, T 26.8%, G 22.7%, A 18.8%
# 0% masked (lower-case)
# ASCII_BASE=33
# EE mean 2.6; min 0.0, lo_quartile 0.6, median 1.5, hi_quartile 3.6, max 24.2

## =======================================================================================
## | A4 | Scout Info
## =======================================================================================

## Read Trimming / Merging Efficency / In-silico PCR

#  0/0  89.23% 858222 (85.8222%)
#  0/5  96.69% 929633 (92.9633%)
#  5/10 96.84% 931586 (93.1586%)
#  5/15 97.05% 933593 (93.3593%)
# 10/10 96.90% 931135 (93.1135%)
# 10/15 97.20% 934016 (93.4016%) <<
# 10/20 96.99% 931865 (93.1865%)
# 15/15 96.96% 931598 (93.1598%)

## =======================================================================================
## | A5 | Primer Error Rate
## =======================================================================================

# Primer - Trimming - Evaluation 
# Mismatch: 0-0 / 515F‐Y / 926R / n: 2328519
# Mismatch: 0-0 / 926R / 515F‐Y / n: 2669
# Mismatch: 0-1 / 515F‐Y / 926R / n: 66869
# Mismatch: 0-1 / 926R / 515F‐Y / n: 79
# Mismatch: 0-2 / 515F‐Y / 926R / n: 8083
# Mismatch: 0-2 / 926R / 515F‐Y / n: 23
# Mismatch: 0-3 / 515F‐Y / 926R / n: 15112 !!!
# Mismatch: 0-3 / 926R / 515F‐Y / n: 4
# Mismatch: 1-0 / 515F‐Y / 926R / n: 50656
# Mismatch: 1-0 / 926R / 515F‐Y / n: 65
# Mismatch: 1-1 / 515F‐Y / 926R / n: 1971
# Mismatch: 1-2 / 515F‐Y / 926R / n: 304
# Mismatch: 1-3 / 515F‐Y / 926R / n: 395
# Mismatch: 2-0 / 515F‐Y / 926R / n: 889
# Mismatch: 2-0 / 926R / 515F‐Y / n: 4
# Mismatch: 2-1 / 515F‐Y / 926R / n: 69
# Mismatch: 2-2 / 515F‐Y / 926R / n: 42
# Mismatch: 2-3 / 515F‐Y / 926R / n: 49
# Mismatch: 3-0 / 515F‐Y / 926R / n: 293
# Mismatch: 3-0 / 926R / 515F‐Y / n: 18
# Mismatch: 3-1 / 515F‐Y / 926R / n: 26
# Mismatch: 3-1 / 926R / 515F‐Y / n: 1
# Mismatch: 3-2 / 515F‐Y / 926R / n: 13
# Mismatch: 3-3 / 515F‐Y / 926R / n: 24
```

## 2.4 B - GZ-to-QC (on scratch)

```
==================================================
Project: p327
Run: run190417
Group: soilclim
N(sample): 96
--------------------------------------------------
START_GZ-2-FQ: 16:43:50 18/04/2019
END_GZ-2-FQ: 16:43:55 18/04/2019
==================================================
```

## 2.5 B - Read-End Trimming and Merging

```
======================================================================
Project: p476
Run: run190923
Group: 16S
N(sample): 96
----------------------------------------------------------------------
Rean-End trimming:
usearch v11.0.667_i86linux64
Trim R1: 10
Trim R2: 15
Read Merging
FLASH v1.2.11
Min Overlap: 15
Max Overlap: 300
Max Mismatch Density: 0.25
----------------------------------------------------------------------
START_SampleResolutionCheck: 13:53:43 23/09/2019
END_SampleResolutionCheck: 13:53:43 23/09/2019
START_Trim_Merge: 13:54:14 23/09/2019
  CH01: N(merged) = 27092 / N(all) = 27915 (Mean Merging Rate: 97.00%)
  CH02: N(merged) = 53644 / N(all) = 55255 (Mean Merging Rate: 97.00%)
  CH03: N(merged) = 41178 / N(all) = 42503 (Mean Merging Rate: 96.00%)
  CH04: N(merged) = 27213 / N(all) = 27955 (Mean Merging Rate: 97.00%)
  CH05: N(merged) = 55270 / N(all) = 56760 (Mean Merging Rate: 97.00%)
  CH06: N(merged) = 30672 / N(all) = 31562 (Mean Merging Rate: 97.00%)
  CH07: N(merged) = 36250 / N(all) = 37244 (Mean Merging Rate: 97.00%)
  CH08: N(merged) = 30481 / N(all) = 31309 (Mean Merging Rate: 97.00%)
  CH09: N(merged) = 24362 / N(all) = 25009 (Mean Merging Rate: 97.00%)
  CH10: N(merged) = 49447 / N(all) = 51018 (Mean Merging Rate: 96.00%)
  CH11: N(merged) = 23927 / N(all) = 24553 (Mean Merging Rate: 97.00%)
  CH12: N(merged) = 70761 / N(all) = 72367 (Mean Merging Rate: 97.00%)
  CH13: N(merged) = 25994 / N(all) = 26699 (Mean Merging Rate: 97.00%)
  CH14: N(merged) = 26493 / N(all) = 27216 (Mean Merging Rate: 97.00%)
  CH15: N(merged) = 22676 / N(all) = 23223 (Mean Merging Rate: 97.00%)
  CH16: N(merged) = 25223 / N(all) = 25934 (Mean Merging Rate: 97.00%)
  CH17: N(merged) = 30741 / N(all) = 31515 (Mean Merging Rate: 97.00%)
  CH18: N(merged) = 34290 / N(all) = 35317 (Mean Merging Rate: 97.00%)
  CH19: N(merged) = 32976 / N(all) = 33910 (Mean Merging Rate: 97.00%)
  CH20: N(merged) = 30370 / N(all) = 31227 (Mean Merging Rate: 97.00%)
  CH21: N(merged) = 32193 / N(all) = 32930 (Mean Merging Rate: 97.00%)
  CH22: N(merged) = 38535 / N(all) = 39415 (Mean Merging Rate: 97.00%)
  CH23: N(merged) = 32829 / N(all) = 33620 (Mean Merging Rate: 97.00%)
  CH24: N(merged) = 24727 / N(all) = 25488 (Mean Merging Rate: 97.00%)
  CH25: N(merged) = 24737 / N(all) = 25435 (Mean Merging Rate: 97.00%)
  CH26: N(merged) = 27803 / N(all) = 28621 (Mean Merging Rate: 97.00%)
  CH27: N(merged) = 22612 / N(all) = 23286 (Mean Merging Rate: 97.00%)
  CH28: N(merged) = 20582 / N(all) = 21154 (Mean Merging Rate: 97.00%)
  CH29: N(merged) = 32921 / N(all) = 33931 (Mean Merging Rate: 97.00%)
  CH30: N(merged) = 25376 / N(all) = 26120 (Mean Merging Rate: 97.00%)
  CH31: N(merged) = 23074 / N(all) = 23739 (Mean Merging Rate: 97.00%)
  CH32: N(merged) = 21717 / N(all) = 22345 (Mean Merging Rate: 97.00%)
  CH33: N(merged) = 27931 / N(all) = 28724 (Mean Merging Rate: 97.00%)
  CH34: N(merged) = 45376 / N(all) = 46645 (Mean Merging Rate: 97.00%)
  CH35: N(merged) = 21357 / N(all) = 21989 (Mean Merging Rate: 97.00%)
  CH36: N(merged) = 29959 / N(all) = 30887 (Mean Merging Rate: 96.00%)
  CH37: N(merged) = 28139 / N(all) = 29008 (Mean Merging Rate: 97.00%)
  CH38: N(merged) = 34090 / N(all) = 34993 (Mean Merging Rate: 97.00%)
  CH39: N(merged) = 36253 / N(all) = 37249 (Mean Merging Rate: 97.00%)
  CH40: N(merged) = 42243 / N(all) = 43448 (Mean Merging Rate: 97.00%)
  CH41: N(merged) = 23538 / N(all) = 24295 (Mean Merging Rate: 96.00%)
  CH42: N(merged) = 31620 / N(all) = 32517 (Mean Merging Rate: 97.00%)
  CH43: N(merged) = 28410 / N(all) = 29367 (Mean Merging Rate: 96.00%)
  CH44: N(merged) = 26929 / N(all) = 27694 (Mean Merging Rate: 97.00%)
  CH45: N(merged) = 23936 / N(all) = 24674 (Mean Merging Rate: 97.00%)
  CH46: N(merged) = 19881 / N(all) = 20565 (Mean Merging Rate: 96.00%)
  CH47: N(merged) = 27243 / N(all) = 28089 (Mean Merging Rate: 96.00%)
  CH48: N(merged) = 24389 / N(all) = 25183 (Mean Merging Rate: 96.00%)
  CH49: N(merged) = 24551 / N(all) = 25238 (Mean Merging Rate: 97.00%)
  CH50: N(merged) = 38153 / N(all) = 39238 (Mean Merging Rate: 97.00%)
  CH51: N(merged) = 37296 / N(all) = 38465 (Mean Merging Rate: 96.00%)
  CH52: N(merged) = 28400 / N(all) = 29241 (Mean Merging Rate: 97.00%)
  CH53: N(merged) = 36280 / N(all) = 37456 (Mean Merging Rate: 96.00%)
  CH54: N(merged) = 29488 / N(all) = 30365 (Mean Merging Rate: 97.00%)
  CH55: N(merged) = 31061 / N(all) = 31940 (Mean Merging Rate: 97.00%)
  CH56: N(merged) = 59239 / N(all) = 60909 (Mean Merging Rate: 97.00%)
  CH57: N(merged) = 26910 / N(all) = 27738 (Mean Merging Rate: 97.00%)
  CH58: N(merged) = 21828 / N(all) = 22485 (Mean Merging Rate: 97.00%)
  CH59: N(merged) = 19166 / N(all) = 19759 (Mean Merging Rate: 96.00%)
  CH60: N(merged) = 25875 / N(all) = 26697 (Mean Merging Rate: 96.00%)
  CH61: N(merged) = 26409 / N(all) = 27250 (Mean Merging Rate: 96.00%)
  CH62: N(merged) = 24952 / N(all) = 25769 (Mean Merging Rate: 96.00%)
  CH63: N(merged) = 26365 / N(all) = 27223 (Mean Merging Rate: 96.00%)
  CH64: N(merged) = 19953 / N(all) = 20580 (Mean Merging Rate: 96.00%)
  CH65: N(merged) = 25865 / N(all) = 26721 (Mean Merging Rate: 96.00%)
  CH66: N(merged) = 36824 / N(all) = 37912 (Mean Merging Rate: 97.00%)
  CH67: N(merged) = 36603 / N(all) = 37767 (Mean Merging Rate: 96.00%)
  CH68: N(merged) = 36757 / N(all) = 37860 (Mean Merging Rate: 97.00%)
  CH69: N(merged) = 19928 / N(all) = 20520 (Mean Merging Rate: 97.00%)
  CH70: N(merged) = 41279 / N(all) = 42543 (Mean Merging Rate: 97.00%)
  CH71: N(merged) = 24353 / N(all) = 25117 (Mean Merging Rate: 96.00%)
  CH72: N(merged) = 29907 / N(all) = 30793 (Mean Merging Rate: 97.00%)
  CH73: N(merged) = 24978 / N(all) = 25782 (Mean Merging Rate: 96.00%)
  CH74: N(merged) = 24477 / N(all) = 25194 (Mean Merging Rate: 97.00%)
  CH75: N(merged) = 17851 / N(all) = 18403 (Mean Merging Rate: 97.00%)
  CH76: N(merged) = 16511 / N(all) = 16930 (Mean Merging Rate: 97.00%)
  CH77: N(merged) = 24644 / N(all) = 25293 (Mean Merging Rate: 97.00%)
  CH78: N(merged) = 20235 / N(all) = 20836 (Mean Merging Rate: 97.00%)
  CH79: N(merged) = 22028 / N(all) = 22617 (Mean Merging Rate: 97.00%)
  CH80: N(merged) = 20405 / N(all) = 21045 (Mean Merging Rate: 96.00%)
  CH81: N(merged) = 14810 / N(all) = 15232 (Mean Merging Rate: 97.00%)
  CH82: N(merged) = 20707 / N(all) = 21281 (Mean Merging Rate: 97.00%)
  CH83: N(merged) = 20190 / N(all) = 20727 (Mean Merging Rate: 97.00%)
  CH84: N(merged) = 31341 / N(all) = 32158 (Mean Merging Rate: 97.00%)
  CH85: N(merged) = 20390 / N(all) = 20930 (Mean Merging Rate: 97.00%)
  CH86: N(merged) = 27874 / N(all) = 28588 (Mean Merging Rate: 97.00%)
  CH87: N(merged) = 25541 / N(all) = 26277 (Mean Merging Rate: 97.00%)
  CH88: N(merged) = 19428 / N(all) = 19960 (Mean Merging Rate: 97.00%)
  CH89: N(merged) = 20109 / N(all) = 20643 (Mean Merging Rate: 97.00%)
  CH90: N(merged) = 23694 / N(all) = 24297 (Mean Merging Rate: 97.00%)
  CH91: N(merged) = 22633 / N(all) = 23166 (Mean Merging Rate: 97.00%)
  CH92: N(merged) = 18135 / N(all) = 18637 (Mean Merging Rate: 97.00%)
  CH93: N(merged) = 14874 / N(all) = 15258 (Mean Merging Rate: 97.00%)
  CH94: N(merged) = 20628 / N(all) = 21200 (Mean Merging Rate: 97.00%)
  CH95: N(merged) = 16343 / N(all) = 16842 (Mean Merging Rate: 97.00%)
  CH96: N(merged) = 19202 / N(all) = 19800 (Mean Merging Rate: 96.00%)
END_Trim_Merge: 13:56:29 23/09/2019
======================================================================
```

## 2.6 C - Primer Trimming with Size Selection

```
==================================================
Project: p476
Run: run190923
Group: 16S
--------------------------------------------------
Primer
515F‐Y: GTGYCAGCMGCCGCGGTAA
926R: CCGYCAATTYMTTTRAGTTT
--------------------------------------------------
usearch v11.0.667_i86linux64
Amplicon range: 100-2000
Number of mis-matches: 1
Coverage: full-length
Wildcards enabled: IUPAC codes
--------------------------------------------------
START_In-Silico_PCR: 14:04:48 23/09/2019
END_In-Silico_PCR: 14:08:12 23/09/2019
==================================================
```

## 2.7 D - Size Selection and Quality Filteringß

```
==================================================
Project: p476
Run: run190923
Group: 16S
--------------------------------------------------
PRINSEQ-lite 0.20.4
Size Range: 200-450
GC Range: 30-70
Min Q Mean: 20
Number of Ns: 0
Low Complexity: dust / 30
--------------------------------------------------
START_QF: 14:08:48 23/09/2019
END_QF: 14:23:44 23/09/2019
--------------------------------------------------
Statistic Report
START_Reporting: 14:23:44 23/09/2019
END_Reporting: 14:25:23 23/09/2019
--------------------------------------------------
Encode Reads
START_Encoding: 14:25:23 23/09/2019
Number of reads: 2490431 / 2490431
END_Encoding: 14:26:23 23/09/2019
--------------------------------------------------
```

Read preparation report:

| Sample | Raw | Merged | Primer | Clean | MeanLength |
| --- | --- | --- | --- | --- | --- |
| CH01 | 27915 | 27092 | 25544 | 25539 | 372.743 |
| CH02 | 55255 | 53644 | 49962 | 49956 | 372.968 |
| CH03 | 42503 | 41178 | 38380 | 38371 | 373.1 |
| CH04 | 27955 | 27213 | 25291 | 25285 | 372.789 |
| CH05 | 56760 | 55270 | 51690 | 51677 | 372.902 |
| CH06 | 31562 | 30672 | 28600 | 28599 | 373.028 |
| CH07 | 37244 | 36250 | 32295 | 32293 | 373.051 |
| CH08 | 31309 | 30481 | 27208 | 27202 | 372.975 |
| CH09 | 25009 | 24362 | 21624 | 21618 | 373.049 |
| CH10 | 51018 | 49447 | 22224 | 22220 | 373.003 |
| CH11 | 24553 | 23927 | 21769 | 21763 | 373.106 |
| CH12 | 72367 | 70761 | 30587 | 30578 | 373.011 |
| CH13 | 26699 | 25994 | 24048 | 24045 | 372.949 |
| CH14 | 27216 | 26493 | 24703 | 24701 | 372.951 |
| CH15 | 23223 | 22676 | 20504 | 20501 | 373.028 |
| CH16 | 25934 | 25223 | 22604 | 22596 | 373.039 |
| CH17 | 31515 | 30741 | 27751 | 27741 | 373.103 |
| CH18 | 35317 | 34290 | 31680 | 31672 | 373.092 |
| CH19 | 33910 | 32976 | 29835 | 29829 | 373.127 |
| CH20 | 31227 | 30370 | 27661 | 27659 | 373.122 |
| CH21 | 32930 | 32193 | 29434 | 29428 | 373.023 |
| CH22 | 39415 | 38535 | 34873 | 34867 | 373.177 |
| CH23 | 33620 | 32829 | 30031 | 30028 | 373.24 |
| CH24 | 25488 | 24727 | 22999 | 22995 | 373.233 |
| CH25 | 25435 | 24737 | 23205 | 23200 | 373.157 |
| CH26 | 28621 | 27803 | 25748 | 25743 | 373.288 |
| CH27 | 23286 | 22612 | 21044 | 21040 | 373.184 |
| CH28 | 21154 | 20582 | 18961 | 18960 | 373.175 |
| CH29 | 33931 | 32921 | 30709 | 30699 | 373.156 |
| CH30 | 26120 | 25376 | 24057 | 24055 | 373.094 |
| CH31 | 23739 | 23074 | 21879 | 21874 | 373.149 |
| CH32 | 22345 | 21717 | 19883 | 19879 | 373.152 |
| CH33 | 28724 | 27931 | 25758 | 25752 | 373.071 |
| CH34 | 46645 | 45376 | 42258 | 42251 | 373.257 |
| CH35 | 21989 | 21357 | 19960 | 19954 | 373.134 |
| CH36 | 30887 | 29959 | 26623 | 26619 | 373.109 |
| CH37 | 29008 | 28139 | 26177 | 26172 | 373.155 |
| CH38 | 34993 | 34090 | 31533 | 31527 | 373.13 |
| CH39 | 37249 | 36253 | 33671 | 33669 | 373.076 |
| CH40 | 43448 | 42243 | 39273 | 39266 | 373.279 |
| CH41 | 24295 | 23538 | 22116 | 22114 | 373.121 |
| CH42 | 32517 | 31620 | 29822 | 29814 | 373.07 |
| CH43 | 29367 | 28410 | 26827 | 26824 | 373.136 |
| CH44 | 27694 | 26929 | 25637 | 25632 | 373.189 |
| CH45 | 24674 | 23936 | 22621 | 22620 | 373.09 |
| CH46 | 20565 | 19881 | 18648 | 18647 | 373.142 |
| CH47 | 28089 | 27243 | 26113 | 26108 | 373.19 |
| CH48 | 25183 | 24389 | 23530 | 23525 | 373.189 |
| CH49 | 25238 | 24551 | 23204 | 23198 | 373.122 |
| CH50 | 39238 | 38153 | 35936 | 35932 | 373.245 |
| CH51 | 38465 | 37296 | 35150 | 35146 | 373.219 |
| CH52 | 29241 | 28400 | 26965 | 26958 | 373.22 |
| CH53 | 37456 | 36280 | 33799 | 33790 | 373.238 |
| CH54 | 30365 | 29488 | 27755 | 27753 | 373.176 |
| CH55 | 31940 | 31061 | 29109 | 29103 | 373.169 |
| CH56 | 60909 | 59239 | 55753 | 55748 | 373.121 |
| CH57 | 27738 | 26910 | 25234 | 25230 | 373.146 |
| CH58 | 22485 | 21828 | 20603 | 20600 | 373.314 |
| CH59 | 19759 | 19166 | 18086 | 18083 | 373.247 |
| CH60 | 26697 | 25875 | 24431 | 24429 | 373.299 |
| CH61 | 27250 | 26409 | 25068 | 25064 | 373.262 |
| CH62 | 25769 | 24952 | 23563 | 23562 | 373.214 |
| CH63 | 27223 | 26365 | 24882 | 24882 | 373.261 |
| CH64 | 20580 | 19953 | 19146 | 19142 | 373.159 |
| CH65 | 26721 | 25865 | 24973 | 24968 | 373.294 |
| CH66 | 37912 | 36824 | 34688 | 34686 | 373.293 |
| CH67 | 37767 | 36603 | 34424 | 34417 | 373.264 |
| CH68 | 37860 | 36757 | 34660 | 34657 | 373.255 |
| CH69 | 20520 | 19928 | 18849 | 18847 | 373.167 |
| CH70 | 42543 | 41279 | 37256 | 37254 | 373.272 |
| CH71 | 25117 | 24353 | 22854 | 22849 | 373.222 |
| CH72 | 30793 | 29907 | 28057 | 28053 | 373.167 |
| CH73 | 25782 | 24978 | 23499 | 23496 | 373.072 |
| CH74 | 25194 | 24477 | 22962 | 22959 | 372.939 |
| CH75 | 18403 | 17851 | 16849 | 16847 | 373.2 |
| CH76 | 16930 | 16511 | 15661 | 15659 | 373.024 |
| CH77 | 25293 | 24644 | 23318 | 23316 | 372.959 |
| CH78 | 20836 | 20235 | 19236 | 19231 | 372.84 |
| CH79 | 22617 | 22028 | 20855 | 20853 | 373.202 |
| CH80 | 21045 | 20405 | 19336 | 19332 | 372.903 |
| CH81 | 15232 | 14810 | 14266 | 14261 | 372.777 |
| CH82 | 21281 | 20707 | 20028 | 20023 | 373.035 |
| CH83 | 20727 | 20190 | 19021 | 19017 | 373.087 |
| CH84 | 32158 | 31341 | 29548 | 29546 | 373.156 |
| CH85 | 20930 | 20390 | 19284 | 19283 | 373.156 |
| CH86 | 28588 | 27874 | 26603 | 26603 | 373.126 |
| CH87 | 26277 | 25541 | 23314 | 23310 | 372.959 |
| CH88 | 19960 | 19428 | 18336 | 18334 | 373.153 |
| CH89 | 20643 | 20109 | 18907 | 18906 | 373.047 |
| CH90 | 24297 | 23694 | 22296 | 22292 | 373.061 |
| CH91 | 23166 | 22633 | 21300 | 21298 | 373.066 |
| CH92 | 18637 | 18135 | 17127 | 17126 | 372.99 |
| CH93 | 15258 | 14874 | 14097 | 14094 | 373.055 |
| CH94 | 21200 | 20628 | 19471 | 19466 | 373.14 |
| CH95 | 16842 | 16343 | 15527 | 15525 | 373.071 |
| CH96 | 19800 | 19202 | 18198 | 18196 | 373.126 |

## 2.8 E - Amplicon Size Variant With Additional Clustering

```
================================================================================
Project: p476
Run: run190923
Group: 16S
File: p476_run190923_16S_bc.fasta
--------------------------------------------------------------------------------
UPARSE & UNOISE3
usearch v11.0.667_i86linux64
--------------------------------------------------------------------------------
START: 14:26:48 23/09/2019
  ▶ Deduplicate Amplicons
  --------------------------------------------------------------------------------
  Dereplicates amplicons to obtain unique amplicons.
  Determin error rates of amplicon reads. 
  --------------------------------------------------------------------------------
  ▶ Cluster OTU (97%)
  --------------------------------------------------------------------------------
  Clusters OTU at 97% using the UPARSE-OTU algorithm.
  Number of OTUs: 8156
  --------------------------------------------------------------------------------
  ▶ Unoise3
  --------------------------------------------------------------------------------
  Uses the UNOISE algorithm to perform denoising (error-correction) of amplicon reads.
  Number of ZOTUs: 13769
  --------------------------------------------------------------------------------
  ▶ Additional Clustering
  --------------------------------------------------------------------------------
  Clusters ZOTUs at different identity levels (i.e. 97%,98% and 99%).
  Number of ZOTUs 99%: 8493
  Number of ZOTUs 98%: 5623
  Number of ZOTUs 97%: 4013
  --------------------------------------------------------------------------------
  ▶ Count Table
  --------------------------------------------------------------------------------
  Generates OTU count tables by mapping reads to OTUs.
  --------------------------------------------------------------------------------
  ▶ ZOTU Table Report
  --------------------------------------------------------------------------------
  Creates a report/summary from an OTU table.
  --------------------------------------------------------------------------------
  ▶ Distance matrices
  --------------------------------------------------------------------------------
  Pairwise distances between (Z)OTU sequences.
  --------------------------------------------------------------------------------
  ▶ Octave plots
  --------------------------------------------------------------------------------
  Octave plots with low-abundance (Z)OTUs and cross-talk information.
  --------------------------------------------------------------------------------
  ▶ Alignment and Tree
  --------------------------------------------------------------------------------
  Creates multiple sequences alignment and clustered tre files.
  The trees will be very approximate in both cases.
  --------------------------------------------------------------------------------
  ▶ Alpha Diversity - Evenness Berger-Parker
  --------------------------------------------------------------------------------
  Evenness based on Berger-Parker.
  A value close to 1 indicates that a single large OTU dominates the sample,
  small values indicate that the reads are distributed over many OTUs.
  --------------------------------------------------------------------------------
  ▶ Rarefaction
  --------------------------------------------------------------------------------
  Calculates a rarefaction curve for an alpha diversity metric.
  The output file can easily be loaded into a spreadsheet or R for generating figures.
  --------------------------------------------------------------------------------
  ▶ Beta Diversity
  --------------------------------------------------------------------------------
  Calculates beta diversity metrics from an OTU table.
  --------------------------------------------------------------------------------
  ▶ Uncross
  --------------------------------------------------------------------------------
  Detects and filters cross-talk (sample mis-assignment) in a OTU table using the UNCROSS algorithm.
  --------------------------------------------------------------------------------
END: 16:51:21 23/09/2019
================================================================================
```

## 2.9 F1 - 16S - Taxonomic Assignment Predictions with SINTAX

```
==========================================================================================
Taxonomic Assignment Predictions with SINTAX and GreenGenes Ref
------------------------------------------------------------------------------------------
Project: p476
Run: run190923
Group: 16S
==========================================================================================
SINTAXv11.0.667_i86linux64
Database: 16S/gg_13_5_utaxformat.fa
Tax filter: 0.85
Workflow Summary:
  (a) Adjust DB according to amplicons (usearch_global; strand both; id 0.7)
  (b) Assign taxa (sintax; strand both)
  (c) Reformat tax information for phyloseq import
  (d) Combine count table and taxa
START_Sintax: 08:27:33 24/09/2019
  Start_F1_trimDB: 08:27:33 24/09/2019
  End_F1_trimDB: 10:51:19 24/09/2019
  Start_F1_Unique_records: 10:51:19 24/09/2019
  End_F1_Unique_records: 10:52:34 24/09/2019
  Start_F1_Build_UPD: 10:52:39 24/09/2019
  End_F1_Build_UPD: 10:53:03 24/09/2019
  Start_F2_for_OTU: 10:53:03 24/09/2019
  End_F2_for_OTU: 11:47:02 24/09/2019
  Start_F2_for_ZOTU: 11:47:02 24/09/2019
  End_F2_for_ZOTU: 15:30:12 24/09/2019
  Start_TaxSummary: 15:30:12 24/09/2019
  End_TaxSummary: 15:30:12 24/09/2019
  Start_ChimeraCheck: 15:30:12 24/09/2019
  End_ChimeraCheck: 15:56:32 24/09/2019
END_Sintax: 15:56:33 24/09/2019
==========================================================================================

==========================================================================================
Taxonomic Assignment Predictions with SINTAX and RDP Ref
------------------------------------------------------------------------------------------
Project: p476
Run: run190923
Group: 16S
==========================================================================================
SINTAXv11.0.667_i86linux64
Database: /16S/RDP_160411/rdp_utaxformat_uniques.fa
Tax filter: 0.85
Workflow Summary:
  (a) Adjust DB according to amplicons (usearch_global; strand both; id 0.7)
  (b) Assign taxa (sintax; strand both)
  (c) Reformat tax information for phyloseq import
  (d) Combine count table and taxa
START_Sintax: 17:47:03 24/09/2019
  Start_F1_trimDB: 17:47:03 24/09/2019
  End_F1_trimDB: 17:52:04 24/09/2019
  Start_F1_Unique_records: 17:52:04 24/09/2019
  End_F1_Unique_records: 17:52:04 24/09/2019
  Start_F1_Build_UPD: 17:52:04 24/09/2019
  End_F1_Build_UPD: 17:52:05 24/09/2019
  Start_F2_for_OTU: 17:52:05 24/09/2019
  End_F2_for_OTU: 17:52:15 24/09/2019
  Start_F2_for_ZOTU: 17:52:15 24/09/2019
  End_F2_for_ZOTU: 17:52:59 24/09/2019
  Start_TaxSummary: 17:52:59 24/09/2019
  End_TaxSummary: 17:52:59 24/09/2019
  Start_ChimeraCheck: 17:52:59 24/09/2019
  End_ChimeraCheck: 17:57:44 24/09/2019
END_Sintax: 17:57:46 24/09/2019
==========================================================================================

==========================================================================================
Taxonomic Assignment Predictions with SINTAX and SILVA Ref
------------------------------------------------------------------------------------------
Project: p476
Run: run190923
Group: 16S
==========================================================================================
SINTAXv11.0.667_i86linux64
Database: 16S/SILVA_128_16S_utax.fasta
Tax filter: 0.85
Workflow Summary:
  (a) Adjust DB according to amplicons (usearch_global; strand both; id 0.7)
  (b) Assign taxa (sintax; strand both)
  (c) Reformat tax information for phyloseq import
  (d) Combine count table and taxa
START_Sintax: 16:51:33 23/09/2019
  Start_F1_trimDB: 16:51:33 23/09/2019
  End_F1_trimDB: 17:47:39 23/09/2019
  Start_F1_Unique_records: 17:47:39 23/09/2019
  End_F1_Unique_records: 17:48:04 23/09/2019
  Start_F1_Build_UPD: 17:48:08 23/09/2019
  End_F1_Build_UPD: 17:48:35 23/09/2019
  Start_F2_for_OTU: 17:48:35 23/09/2019
  End_F2_for_OTU: 18:58:49 23/09/2019
  Start_F2_for_ZOTU: 18:58:50 23/09/2019
  End_F2_for_ZOTU: 23:31:00 23/09/2019
  Start_TaxSummary: 23:31:00 23/09/2019
  End_TaxSummary: 23:31:00 23/09/2019
  Start_ChimeraCheck: 23:31:00 23/09/2019
  End_ChimeraCheck: 23:57:39 23/09/2019
END_Sintax: 23:57:41 23/09/2019
==========================================================================================
```

## 2.10 G1 - Summary 16S

```
## (Z)OTU Summary:

# Number of OTUs:       8,156
# Number of ZOTUs:     13,769
# Number of ZOTUs 99%:  8,493
# Number of ZOTUs 98%:  5,623
# Number of ZOTUs 97%:  4,013

## Annotation Level Summary:

# OTU:
# k:  30
# d: * 96
# p:  53
# c: * 80
# o: * 136
# f: ****** 670
# g: ************************************************************ 7091

# ZOTU:
# k: * 92
# d: *** 226
# p: ********************* 1603
# c: *************** 1099
# o: ************************ 1813
# f: ************************************************************ 4516
# g: *********************************************************** 4420

# ZOTU_c99:
# k: * 69
# d: *** 146
# p: ******************* 907
# c: ************** 658
# o: ************************ 1169
# f: ******************************************************** 2668
# g: ************************************************************ 2876

# ZOTU_c98:
# k: ** 58
# d: *** 105
# p: **************** 544
# c: ************* 446
# o: *********************** 762
# f: ************************************************** 1679
# g: ************************************************************ 2029

# ZOTU_c97:
# k: ** 53
# d: *** 82
# p: ************* 342
# c: ************* 325
# o: ******************** 515
# f: ******************************************** 1146
# g: ************************************************************ 1550
```

## 2.11 F2 - ITS - Taxonomic Assignment Predictions

```
==========================================================================================
Taxonomic Assignment Predictions with SINTAX
------------------------------------------------------------------------------------------
Project: p327
Run: run190417
Group: ITS
==========================================================================================
SINTAXv11.0.667_i86linux64
Database: ITS/UNITE_UTAX_V7.2_10.10.2017.fasta
Tax filter: 0.85
Workflow Summary:
 (a) Adjust DB according to amplicons (usearch_global; strand both; id 0.7)
 (b) Assign taxa (sintax; strand both; sintax_cutoff 0.7)
 (c) Reformat tax information for phyloseq import
 (d) Combine count table and taxa
START_Sintax: 15:48:58 02/05/2019
 Start_F1_trimDB: 15:48:58 02/05/2019
 End_F1_trimDB: 16:20:16 02/05/2019
 Start_F1_Unique_records: 16:20:16 02/05/2019
 End_F1_Unique_records: 16:20:19 02/05/2019
 Start_F1_FilterG: 16:20:19 02/05/2019
 End_F1_FilterG: 16:20:20 02/05/2019
 Start_F1_Build_UPD: 16:20:20 02/05/2019
 End_F1_Build_UPD: 16:20:28 02/05/2019
 Start_F2_for_OTU: 16:20:28 02/05/2019
 End_F2_for_OTU: 16:20:55 02/05/2019
 Start_F2_for_ZOTU: 16:20:55 02/05/2019
 End_F2_for_ZOTU: 16:21:31 02/05/2019
 Start_TaxSummary: 16:21:31 02/05/2019
 End_TaxSummary: 16:21:31 02/05/2019
 Start_ChimeraCheck: 16:21:31 02/05/2019
 End_ChimeraCheck: 16:24:11 02/05/2019
END_Sintax: 16:24:11 02/05/2019
==========================================================================================
```

## 2.12 G2 - Summary ITS

```
## (Z)OTU Summary:

# Number of OTUs:       2277
# Number of ZOTUs:      1164
# Number of ZOTUs 99%:   851
# Number of ZOTUs 98%:   781
# Number of ZOTUs 97%:   730

## Annotation Level Summary:

# OTU:
# d: ********************** 212
# p: ************************************************************ 582
# c: ************************************************ 466
# o: *********** 110
# f: ************ 113
# g: ***************************************** 397
# s: ***************************************** 397

# ZOTU:
# d: ************************************************************ 545
# p: ******* 62
# c: *********** 97
# o: **************** 144
# f: ************* 117
# g: **************** 143
# s: ****** 56

# ZOTU_c99:
# d: ************************************************************ 397
# p: ******* 49
# c: ********** 69
# o: *************** 101
# f: ************ 80
# g: ***************** 110
# s: ******* 45

# ZOTU_c98:
# d: ************************************************************ 371
# p: ****** 40
# c: *********** 70
# o: *************** 91
# f: *********** 67
# g: **************** 100
# s: ******* 42

# ZOTU_c97:
# d: ************************************************************ 344
# p: ******* 40
# c: ************ 66
# o: *************** 88
# f: ********** 60
# g: ***************** 96
# s: ****** 36
```

Note: Problems with annotation - not all OTUs are fungi related but eukaryotes instead: e.g.: ZOTU2 k\_\_Eukaryota; p\_\_Streptophyta; c\_\_Liliopsida; o\_\_Poales; f\_\_Poaceae; g\_\_

We removed all “Eukaryota” related ZOTUs

## 2.13 ITSx to identify (more) non-fungi related ZOTUs

> ITSx (Bengtsson-Palme et al. 2013 - https://microbiology.se/software/itsx/)

```
Version: itsx 1.1.1

Number of sequences in input file:       851
Sequences detected as ITS by ITSx:       850
 On main strand:                        778
 On complementary strand:                72
Sequences detected as chimeric by ITSx:    0
ITS sequences by preliminary origin:
 A Alveolates:          109
 D Amoebozoa:           4
 C Bacillariophyta:     0
 I Brown algae:         0
 B Bryophytes:          14
 E Euglenozoa:          3
 U Eustigmatophytes:    3
 F Fungi:               520
 G Green algae:         70
 L Liverworts:          0
 M Metazoa:             41
 ? Microsporidia:       0
 O Oomycetes:           7
 P Prymnesiophytes:     0
 Q Raphidophytes:       0
 H Red algae:           0
 R Rhizaria:            29
 S Synurophyceae:       6
 T Tracheophyta:        44
```
